# pH-Dependence of the Aqueous Phase Room Temperature Brønsted Acid-Catalyzed Chemoselective Oxidation of Sulfides with H_2_O_2_

**DOI:** 10.3390/molecules200916709

**Published:** 2015-09-14

**Authors:** Hai-Min Shen, Wen-Jie Zhou, Xin Ma, Hong-Ke Wu, Wu-Bin Yu, Ning Ai, Hong-Bing Ji, Hong-Xin Shi, Yuan-Bin She

**Affiliations:** 1 College of Chemical Engineering, Zhejiang University of Technology, Hangzhou 310014, China; E-Mails: zhouwj0716@163.com (W.-J.Z.); shm190726@hotmail.com (X.M.); wuhongke@zjut.edu.cn (H.-K.W.); yuwb@zjut.edu.cn (W.-B.Y.); aining@zjut.edu.cn (N.A.); shihxin@zjut.edu.cn (H.-X.S.); 2 School of Chemistry and Chemical Engineering, Sun Yat-Sen University, Guangzhou 510275, China; E-Mail: jihb@mail.sysu.edu.cn

**Keywords:** sulfide, oxidation, sulfoxide, pH-dependence, Brønsted acid

## Abstract

A pH-dependence of the Brønsted acid-catalyzed oxidation of sulfides to the corresponding sulfoxides with H_2_O_2_ is reported for the first time based on our systematic investigation of the catalytic performance of a series of Brønsted acids. For all of the Brønsted acids investigated, the catalytic performances do not depend on the catalyst loading (mol ratio of Brønsted acid to substrate), but rather depend on the pH value of the aqueous reaction solution. All of them can give more than 98% conversion and selectivity in their aqueous solution at pH 1.30, no matter how much the catalyst loading is and what the Brønsted acid is. This pH-dependence principle is a very novel perspective to understand the Brønsted-acid catalysis system compared with our common understanding of the subject.

## 1. Introduction

The chemoselective oxidation of sulfides to the corresponding sulfoxides is a very important transformation in organic synthesis, since the obtained sulfoxides are important intermediates or building blocks in the construction of pharmaceuticals, agrochemicals, and other valuable fine chemicals [1,2,3,4,5,6,7]. To realize this extremely useful transformation, several oxidants can be employed, such as hydrogen peroxide [8,9,10,11,12,13], molecular oxygen [14,15,16,17,18,19], *tert*-butyl hydroperoxide [20,21,22,23], cumene hydroperoxide [24,25,26,27], iodobenzene diacetate [28,29,30], sodium hypochlorite [31,32,33], and oxone [34]. Among these oxidants, aqueous hydrogen peroxide is a very promising reagent compared to others, as it is readily available, inexpensive, easy to handle, can be safely stored, and it is environmentally benign with harmless water being the only by product generated in the reaction [9,35,36]. However, the oxidation of sulfides to sulfoxides employing aqueous hydrogen peroxide in the absence of catalyst is a very slow process, especially at room temperature. Thus, in order to accelerate this chemical transformation, several catalytic systems have been developed such as transition metal complexes catalysis [37,38,39,40,41], Brønsted-acid catalysis [35,42,43,44,45], organocatalyst catalysis [10,46,47,48,49,50,51,52,53,54,55], metal and metal oxide nanoparticles catalysis [56,57,58], heteropoly acid catalysis [13,59,60,61], and the heterogenization of the above catalytic systems [36,58,62,63,64,65,66]. Considering the high cost of the metal catalysts, inevitable metal residue contamination of the products, and the toxicity of the transition metal complexes, Brønsted acids and organocatalysts seem to be the more attractive choices, as well as for the high catalytic activity and selectivity seen in Brønsted acids and organocatalysts catalysis.

Several Brønsted acids have been employed in the Brønsted-acid catalysis system to catalyze the chemoselective oxidation of sulfides to corresponding sulfoxides, such as sulfuric acid (H_2_SO_4_) [62], hydrochloric acid (HCl) [62], nitric acid (HNO_3_) [67], boric acid (H_3_BO_3_) [35], chiral phosphoric acid [68], acetic acid (CH_3_COOH) [45], oxalic acid (HOOCCOOH) [44], *p*-toluenesulfonic acid (H_3_CC_6_H_4_SO_3_H) [69], sulfamic acid (H_2_NSO_3_H) [43], dodecyl hydrogen sulfate [42], immobilized sulfuric acid [62], immobilized phosphoric acid [70], immobilized sulfamic acid [36], and immobilized vanadic acid [71].Excellent catalytic activity and chemoselectivity, and even enantioselectivity, could usually be obtained in the above catalysis systems by optimizing the catalyst loading (mol ratio of Brønsted acid to substrate). Although so many Brønsted-acid catalysis systems have been successfully introduced in the chemoselective oxidation of sulfides to corresponding sulfoxides, a common feature of all these different Brønsted-acid catalysis systems is that by far they have not been investigated systematically.

In our screening of modifying groups for β-cyclodextrin to construct artificial enzymes [72,73,74], especially sulfide-oxidase, we found that the conversion, yield and chemoselectivity in the Brønsted acid-catalyzed oxidation of sulfides seemed to be dependent on the catalyst loading, which meant that different Brønsted acids could exhibit their optimal catalytic activity by optimizing its catalyst loading for most cases, but in fact they depended on the concentration of the Brønsted acid in the aqueous phase, no matter how much the catalyst loading was. These findings inspired us to try to explore some commonness in the Brønsted acid-catalyzed oxidation of sulfides with H_2_O_2_, and finally a pH-dependence principle was obtained. In this paper, we disclose the pH-dependence principle of the Brønsted acid-catalyzed chemoselective oxidation of sulfides with H_2_O_2_ based on our systematic investigation of the catalytic performance of a series of Brønsted acids, in which the conversion, yield and chemoselectivity depended only on the pH of the aqueous reaction solution, no matter how much the catalyst loading is and what the Brønsted acid is. To the best of our knowledge, our study reveals a commonness in the Brønsted acid-catalyzed oxidation of sulfides with H_2_O_2_ for the first time, so these attempts to explore the commonness in the different Brønsted acid-catalyzed chemoselective oxidation of sulfides with H_2_O_2_ will not only provide a summary of the abundant previous studies, but also an important reference for further studies of the Brønsted acid-catalyzed oxidation of sulfides, and even other Brønsted-acid catalysis systems.

## 2. Results and Discussion

Camphor-10-sulfonic acid derived from the natural product camphor was the first Brønsted acid investigated in our research. In order to optimize the reaction conditions, we chose the catalytic oxidation of methyl phenyl sulfide as a probe reaction and systematically evaluated the effect of catalyst loading on the probe reaction. As shown in Table 1, with the increase of catalyst loading from 0.0% to 25.0%, the conversion and yield of the probe reaction increased from 34.1% to 99.2% and 34.1% to 98.3% respectively, and meanwhile the chemoselectivity remained above 99.0% in cases. It seems that the conversion and yield of the probe reaction depend heavily on the catalyst loading, but when we increased the amount of substrate methyl phenyl sulfide from 1.0 mmol to 10.0 mmol and kept the other reaction conditions as the same as Entry 11 in Table 1, which meant that the catalyst loading decreased from 25.0% to 2.5%, surprisingly, the conversion and yield of the probe reaction almost did not decrease as expected, and the conversion, yield and selectivity remained at the same level, as shown in Supplementary Materials Table S1. When we decreased the amount of methyl phenyl sulfide substrate from 1.0 mmol to 0.40 mmol and kept the other reaction conditions as the same as Entry 2 in Table 1, which meant that the catalyst loading increased from 2.5% to 6.25%, the conversion and yield of the probe reaction did not increase as expected either, as shown in Supplementary Materials Table S2. These findings inspired us to consider that in the Brønsted acid-catalyzed chemoselective oxidation of sulfides to the corresponding sulfoxides, the conversion and yield might not depend on the catalyst loading, but rather on the concentration of Brønsted acid in the aqueous phase.

In order to verify the generality of our findings, the scope of Brønsted acids were extended from camphor-10-sulfonic acid to methanesulfonic acid, *p*-toluenesulfonic acid, methanoic acid and acetic acid. The experimental data is presented in the Supplementary Materials. It is very clear that the relationships between conversion and yield to catalyst loading in these typical Brønsted-acid catalysis systems are as the same as with camphor-10-sulfonic acid. The conversion and yield seem to be dependent on the catalyst loading, but in fact depend on the concentration of Brønsted acid in the aqueous phase. It was also obtained from the experimental data that, to achieve their optimal catalytic performances, catalyst concentrations may be different for different Brønsted acids. In our research, the amount of solvent was fixed at 2.0 mL, but to achieve their optimized catalytic performances, the amounts of Brønsted acid needed were 0.25, 0.25, 0.25, 4.00 and 5.50 mmol for camphor-10-sulfonic acid, methanesulfonic acid, *p*-toluenesulfonic acid, methanoic acid and acetic acid, respectively, as shown in Table 2. Thus, it could be concluded that in the Brønsted acid-catalyzed chemoselective oxidation of sulfides to the corresponding sulfoxides, the catalytic performance depended on the concentration of Brønsted acid in the aqueous phase, and different Brønsted acid exhibited optimized catalytic performance at different catalyst concentrations.

**Table 1:** Effect of catalyst loading on the oxidation of methyl phenyl sulfide with H_2_O_2_ catalyzed by camphor-10-sulfonic acid ^a^. 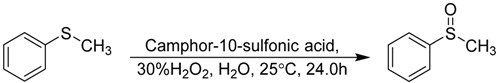

Entry	Catalyst Loading (%)	Conversion (%) ^b^	Yield (%) ^b^
1	0.0	34.1	34.1
2	2.5	41.6	41.5
3	5.0	51.8	51.5
4	7.5	56.2	56.0
5	10.0	59.5	59.2
6	12.5	64.4	64.0
7	15.0	79.4	78.8
8	17.5	84.5	83.9
9	20.0	93.4	92.7
10	22.5	97.6	96.8
11	25.0	99.2	98.3

^a^ Reaction conditions: substrate (1.0 mmol, 124.2 mg), 30% H_2_O_2_ (1.15 mmol, 130 µL), solvent (2.0 mL H_2_O), 25 °C, 24.0 h; ^b^ Conversion and selectivity were determined by GC with *p*-xylene as internal standard, product was determined by comparison with the authentic sample.

**Table 2:** Optimized amount of Brønsted acid-catalysts in the oxidation of methyl phenyl sulfide with H_2_O_2_^a^ and the pH value of the aqueous reaction solution. 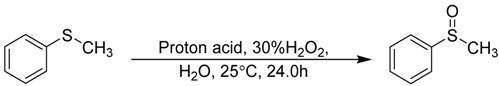

Entry	Brønsted Acid	Amount (mmol)	pH Value ^b^	Conversion (%) ^c^	Yield (%) ^c^
1	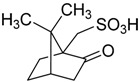	0.25	1.28	99.6	99.3
2	CH_3_SO_3_H	0.25	1.25	99.7	98.8
3	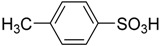	0.25	1.30	99.7	98.8
4	HCOOH	4.00	1.31	99.1	98.6
5	CH_3_COOH	5.50	1.35	99.1	98.9

^a^ Reaction conditions: substrate (1.0 mmol, 124.2 mg), 30% H_2_O_2_ (1.15 mmol, 130 µL), solvent (2.0 mL H_2_O), 25 °C, 24.0 h; ^b^ pH of the solutions were measured independently without substrate and oxidant; ^c^ Conversion and selectivity were determined by GC with *p*-xylene as internal standard, product was determined by comparison with the authentic sample.

In order to further confirm this commonness in the Brønsted acid-catalyzed oxidation of sulfides to the corresponding sulfoxides, some shared features in the aqueous solution reactions in Table 2 must be explored. As a quantitative index for the aqueous solution of Brønsted acid, the pH values of the aqueous reaction solutions in Table 2 were measured by a pH meter and are shown in Table 2. To our surprise they were similar, so we speculate that the common feature in the different Brønsted acid-catalyzed chemoselective oxidations of sulfides might be the same pH level, no matter what the Brønsted acid is.

To verify our speculation, several typical Brønsted acids, such as camphor-10-sulfonic acid, methanesulfonic acid, *p*-toluenesulfonic acid, methanoic acid, sulfuric acid, hydrochloric acid, trifluoroacetic acid and boric acid were selected to prepare aqueous solutions with a series of pH values, and their catalytic performance in the probe reaction were investigated systematically. As shown in Table 3, in the aqueous solution of camphor-10-sulfonic acid, when the pH value decreased from 2.00 to 1.30, the conversion and yield of the probe reaction increased from 42.6% to 99.6% and 42.6% to 98.9%, respectively, and the chemoselectivity stayed at around 99.0%. 

**Table 3:** Effect of pH value on the oxidation of methyl phenyl sulfide with H_2_O_2_ catalyzed by camphor-10-sulfonic acid ^a^. 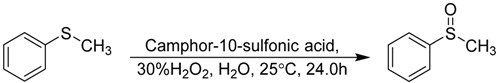

Entry	pH Value ^b^	Conversion (%) ^c^	Yield (%) ^c^
1	2.00	42.6	42.6
2	1.90	49.5	49.2
3	1.80	58.1	57.9
4	1.70	66.9	66.4
5	1.60	72.1	71.4
6	1.50	77.9	77.1
7	1.40	98.1	96.3
**8**	**1.30**	**99.6**	**98.9**
9	1.20	99.8	98.6
10	1.10	99.6	97.2
11	1.00	99.4	96.5

^a^ Reaction conditions: substrate (1.0 mmol, 124.2 mg), 30% H_2_O_2_ (1.15 mmol, 130 µL), catalyst and solvent (2.0 mL aqueous solution of camphor-10-sulfonic acid), 25 °C, 24.0 h; ^b^ pH of the solutions were measured independently without substrate and oxidant; ^c^ Conversion and selectivity were determined by GC with *p*-xylene as internal standard, product was determined by comparison with the authentic sample.

A further decrease in the pH value led to no increase in all of the conversion, yield and selectivity, but rather a slight decrease. Similar catalytic performance profiles with pH of the aqueous solution reaction were obtained for all of the Brønsted-acid catalysis systems employed herein. The experimental data is presented in the Supplementary Materials. All of them exhibited their optimized catalytic performance when the pH value of the aqueous reaction solution was 1.30. When we increased the amount of methyl phenyl sulfide from 1.0 to 10.0 mmol, and the other reaction conditions were kept as the same as Entry 8 in Table 3, all of the conversions, yield and selectivity stayed at the same level as expected, without any decrease, as shown in Supplementary Materials Table S3. Thus, it is very obvious that in the Brønsted acid-catalyzed oxidation of sulfides to the corresponding sulfoxides, pH-dependence is a common feature, and no matter which Brønsted acids are used, they can all achieve their optimal catalytic performance in their aqueous solution of pH 1.30, and the catalytic performance is not dependent on the catalyst loading which is a very novel perspective to understand the Brønsted-acid catalysis system compared with our common knowledge of this process.

In order to verify the generality of the pH-dependence principle, both the scope of Brønsted acids and sulfides were extended. It is clear from Table 4 that, for all of the typical Brønsted acids investigated, satisfactory conversion, yield and selectivity could be obtained in their aqueous reaction solution of pH 1.30, and the conversion, yield and selectivity fluctuate in a very small range, being nearly constant. 

**Table 4:** Oxidation of methyl phenyl sulfide with H_2_O_2_ catalyzed by Brønsted acids atpH 1.30 ^a,b^. 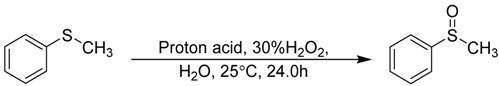

Entry	Brønsted Acid	Conversion (%) ^c^	Yield (%) ^c^
1	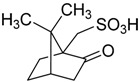	99.6	98.9
2	CH_3_SO_3_H	99.8	98.7
3	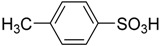	99.7	98.5
4	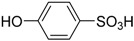	98.7	97.9
5	HCOOH	99.6	99.1
6	CH_3_COOH	99.2	98.6
7	HOOCCOOH	99.7	98.3
8	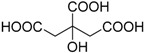	99.8	98.5
9	CF_3_COOH	98.8	97.7
10	H_2_SO_4_	99.6	99.1
11	HCL	99.7	98.8
12	H_3_PO_4_	99.5	98.9
13	H_3_BO_3_	99.6	98.7
14	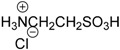	99.6	98.8
15	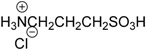	99.7	98.5

^a^ Reaction conditions: substrate (1.0 mmol, 124.2 mg), 30% H_2_O_2_ (1.15 mmol, 130 µL), catalyst and solvent (2.0 mL pH 1.30 aqueous solution of Brønsted acid), 25 °C, 24.0 h; ^b^ pH of the solutions were measured independently without substrate and oxidant; ^c^ Conversion and selectivity were determined by GC with *p*-xylene as internal standard, product was determined by comparison with the authentic sample.

When the scope of sulfides was extended to a series of structurally diverse sulfides, a good substrate tolerance was observed when employing camphor-10-sulfonic acid as a typical Brønsted acid catalyst in pH 1.30 aqueous solution. As shown in Table 5, most of the sulfides could be oxidized to the corresponding sulfoxides in more than 98% conversion and selectivity, especially for some liquid sulfides because of their excellent dispersion in water. As for sulfides with poor water solubility, a mixture of H_2_O and CH_3_CN in the volume ratio of 1:1 was employed as solvent to enhance the substrate dispersion, and satisfactory catalytic performance was achieved too.

**Table 5:** Oxidation of various sulfides to sulfoxides with 30% H_2_O_2_ catalyzed by camphor-10-sulfonic acid in pH 1.30 ^a,b^.

Entry	Sulfide	Conversion (%) ^c^	Yield (%) ^c^
1	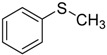	99.6	98.9
2	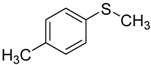	98.7	97.9
3	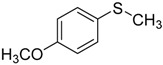	99.9	99.4
4	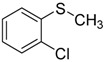	99.4	98.8
5 ^d^	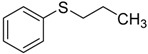	98.5	97.6
6 ^d^	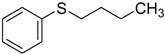	98.1	97.4
7 ^d^	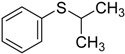	98.7	98.2
8	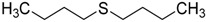	99.5	99.1
9	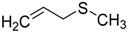	99.9	99.4
10 ^d^	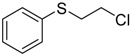	98.7	97.9
11 ^d^	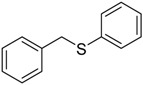	96.8	95.6
12	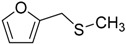	99.2	98.6

^a^ Reaction conditions: substrate (1.0 mmol), 30% H_2_O_2_ (1.15 mmol, 130 µL), catalyst and solvent (2.0 mL pH 1.30 aqueous solution of camphor-10-sulfonic acid), 25 °C, 24.0 h; ^b^ pH of the solutions were measured independently without substrate and oxidant; ^c^ Conversion and selectivity were determined by GC or HPLC with *p*-xylene as internal standard, product was determined by comparison with the authentic sample synthesized following the literature procedure [75] and characterized by ^1^H-NMR, ^13^C-NMR and ESI-MS; ^d^ H_2_O and CH_3_CN in the volume ratio of 1:1 as solvent.

## 3. Experimental Section

### 3.1. General Information

^1^H-NMR and ^13^C-NMR spectra were measured on a AVANCE III NMR spectrometer (Bruker, Fällanden, Switzerland) at 400 MHz in CDCl_3_. Tetramethylsilane was used as the internal standard (0.00 ppm) in CDCl_3_. The ESI-MS spectra were measured on an Agilent 6210 TOF LC/MS mass spectrometer (Agilent Technologies, Palo Alto, CA, USA). GC analyses were performed on a GC-14B instrument (Shimadzu, Kyoto, Japan) using a SPB-5 column (0.25 mm × 30 m). HPLC analyses were performed on a Waters 1525 chromatograph (Waters, Milford, MA, USA) equipped with a Waters 2996 Photodiode Array Detector using a Hypersil ODS2-5 column (4.6 mm × 250 mm). All the catalytic reaction were carried out in the Wattecs parallel synthesis system (Wattecs, Xi’an, China).

### 3.2. Materials

Sulfides used were purchased from Shanghai Energy Chemical Co. Ltd. (Shanghai, China), Aladdin Chemistry Co. Ltd. (Shanghai, China), TCI Co. Ltd. (Tokyo, Japan), Aldrich Co. Ltd. (Shanghai, China), or synthesized from thiophenol and corresponding alkyl bromide. Methyl phenyl sulfoxide was purchased from Aldrich Co. Ltd. (Shanghai, China). Other sulfoxides were synthesized following the literature procedure [75] and characterized by ^1^H-NMR, ^13^C-NMR and ESI-MS. Camphor-10-sulfonic acid, methanesulfonic acid, *p*-toluenesulfonic acid, methanoic acid, sulfuric acid, hydrochloric acid, trifluoroacetic acid, boric acid, taurine (2-aminoethanesulfonic acid), homotaurine (3-aminopropanesulfonic acid) were purchased from Shanghai Energy Chemical Co. Ltd., Aladdin Chemistry Co. Ltd., and TCI Co. Ltd. 30% H_2_O_2_ were purchased from Shanghai Lingfeng Chemical Reagent Co. Ltd. (Shanghai, China), All the other common reagents were of an analytical grade. All of the reagents were used as received without further purification unless otherwise noted.

### 3.3. General Procedure for Oxidation of Sulfides Catalyzed by Brønsted Acid (1)

Sulfide (1.0 mmol) was added to a solution of Brønsted acid in water (2.0 mL) in corresponding amount, after stirring at 25 °C for 15 min, 30% H_2_O_2_ (1.15 mmol) was added. Then, the resultant mixture was kept stirring at 25 °C for 24.0 h. At the end of the reaction, saturated aqueous Na_2_SO_3_ (2.0 mL) was added to stop deep oxidation. The obtained solution was extracted with ethyl acetate (3 × 2.0 mL), and the combined organic phase was washed with brine (2.0 mL) and dried over anhydrous Na_2_SO_4_. The obtained solution was analyzed by GC or HPLC to determine the conversion and yield with *p*-xylene as internal standard.

### 3.4. General Procedure for Oxidation of Sulfides Catalyzed by Brønsted Acid (2)

Sulfide (1.0 mmol) was added to a aqueous solution of Brønsted acid in certain pH (2.0 mL), after stirring at 25 °C for 15 min, 30% H_2_O_2_ (1.15 mmol) was added. Then, the resultant mixture was kept stirring at 25 °C for 24.0 h. At the end of the reaction, saturated aqueous Na_2_SO_3_ (2.0 mL) was added to stop deep oxidation. The obtained solution was extracted with ethyl acetate (3 × 2.0 mL), and the combined organic phase was washed with brine (2.0 mL) and dried over anhydrous Na_2_SO_4_. The obtained solution was analyzed by GC or HPLC to determine the conversion and yield with *p*-xylene as internal standard.

## 4. Conclusions

In conclusion, a pH-dependence principle in Brønsted acid-catalyzed chemoselective oxidation of sulfides to the corresponding sulfoxides with H_2_O_2_ is reported for the first time. For all the Brønsted acids investigated, their catalytic performances depend on the pH value of the aqueous solution reaction, and do not depend on the Brønsted acid loading (mol ratio of Brønsted acid to substrate). All of the Brønsted acids investigated can give more than 98% conversion and selectivity in their aqueous solution of pH 1.30 with an excellent substrate tolerance, no matter what the Brønsted acids are. To the best of our knowledge, this pH-dependence principle is the first common feature reported in the Brønsted acid-catalyzed chemoselective oxidation of sulfides and represents a very novel perspective to understand the Brønsted-acid catalysis system compared with our common understanding of these reactions. Thus, our finding will be an important reference not only to further studies of Brønsted acid-catalyzed oxidations of sulfides, but also to other Brønsted-acid catalysis systems. Further investigation on the catalytic mechanism and attempts to apply the pH-dependence principle to other Brønsted acid-catalyzed systems are under way.

## Supplementary Materials

Supplementary materials can be accessed at: http://www.mdpi.com/1420-3049/20/09/16709/s1.
