# Supplementary material for: pH-Dependence of the Aqueous Phase Room Temperature Brønsted Acid-Catalyzed Chemoselective Oxidation of Sulfides with H2O2
Source: Molecules. 2015 Sep 14;20(9):16709–22. doi: 10.3390/molecules200916709 (PMC6331969; doi:10.3390/molecules200916709)
Supplement: Supplementary file 1 [file molecules-20-16709-s001.pdf]

Supplementary Materials

1. Experimental Data

Table S1. Effect of the substrate amount on the oxidation of methyl phenyl sulfide with H_2O_2 catalyzed by camphor-10-sulfonic acid [a].

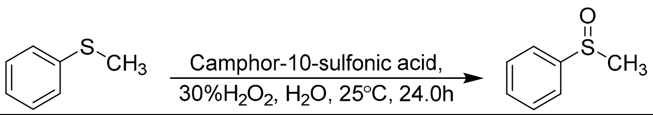

Entry	Substrate Concentration (mmol/mL)	Conversion (%) [b]	Yield (%) [b]
1	0.5	99.6	99.3
2	1.0	99.8	99.0
3	2.0	99.7	97.7
4	3.0	99.7	97.8
5	4.0	99.9	97.5
6	5.0	99.9	98.3

[a] Reaction conditions: catalyst camphor-10-sulfonic acid (0.25 mmol, 58.1 mg), 30% H_2O_2 (1.15 equ.), solvent (2.0 mL H_2O), 25 °C, 24.0 h; [b] Conversion and selectivity were determined by GC with *p*-xylene as internal standard, product was determined by comparison with the authentic sample.

Table S2. Effect of the substrate amount on the oxidation of methyl phenyl sulfide with H_2O_2 catalyzed by camphor-10-sulfonic acid [a].

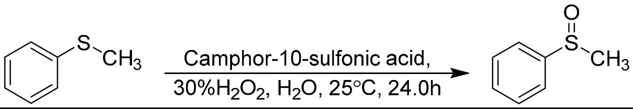

Entry	Substrate Concentration (mmol/mL)	Conversion (%) [b]	Yield (%) [b]
1	0.5	39.8	39.8
2	0.4	38.6	38.6
3	0.3	43.6	43.6
4	0.2	46.4	46.4

[a] Reaction conditions: catalyst camphor-10-sulfonic acid (0.025 mmol, 5.8 mg), 30% H_2O_2 (1.15 equ.), solvent (2.0 mL H_2O), 25 °C, 24.0 h; [b] Conversion and selectivity were determined by GC with *p*-xylene as internal standard, product was determined by comparison with the authentic sample.

Table S3. Effect of the substrate amount on the oxidation of methyl phenyl sulfide with H_2O_2 catalyzed by camphor-10-sulfonic acid in pH 1.30 [a].

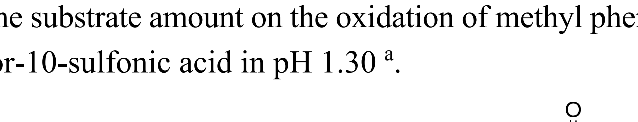

Entry	Substrate Concentration (mmol/mL)	Conversion (%) [b]	Yield (%) [b]
1	0.5	99.8	98.3
2	1.0	99.7	98.0
3	2.0	99.7	97.0
4	3.0	99.8	97.4
5	4.0	99.8	97.4
6	5.0	99.9	97.6

[a] Reaction conditions: 30% H_2O_2 (1.15 equ.), catalyst and solvent (2.0 mL pH 1.30 aqueous solution of camphor-10-sulfonic acid), 25 °C, 24.0 h; [b] Conversion and selectivity were determined by GC with *p*-xylene as internal standard, product was determined by comparison with the authentic sample.

Table S4. Effect of catalyst loading on the oxidation of methyl phenyl sulfide with H_2O_2 catalyzed by methanesulfonic acid [a].

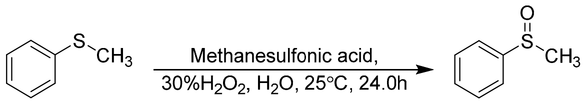

Entry	Catalyst Loading (%)	Conversion (%) [b]	Yield (%) [b]
1	0.0	36.5	36.5
2	2.5	39.0	39.0
3	5.0	48.9	48.9
4	7.5	53.8	53.6
5	10.0	66.0	65.6
6	12.5	68.2	67.8
7	15.0	77.6	77.1
8	17.5	84.3	83.7
9	20.0	90.0	89.2
10	22.5	97.4	96.6
**11**	**25.0**	**99.1**	**98.5**

[a] Reaction conditions: substrate (1.0 mmol, 124.2 mg), 30% H_2O_2 (1.15 mmol, 130 μL), solvent (2.0 mL H_2O), 25 °C, 24.0 h; [b] Conversion and selectivity were determined by GC with *p*-xylene as internal standard, product was determined by comparison with the authentic sample.

Table S5. Effect of the substrate amount on the oxidation of methyl phenyl sulfide with H_2O_2 catalyzed by methanesulfonic acid [a].

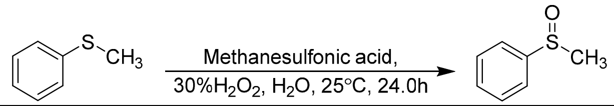

Entry	Substrate Concentration (mmol/mL)	Conversion (%) [b]	Yield (%) [b]
1	0.5	99.7	98.8
2	1.0	99.8	98.4
3	2.0	99.9	98.0
4	3.0	99.8	97.7
5	4.0	99.9	97.5
6	5.0	99.9	97.7

[a] Reaction conditions: catalyst methanesulfonic acid (0.25 mmol, 24.0 mg), 30% H_2O_2 (1.15 equ.), solvent (2.0 mL H_2O), 25 °C, 24.0 h; [b] Conversion and selectivity were determined by GC with *p*-xylene as internal standard, product was determined by comparison with the authentic sample.

Table S6. Effect of the substrate amount on the oxidation of methyl phenyl sulfide with H_2O_2 catalyzed by methanesulfonic acid [a].

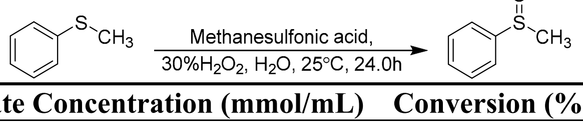

Entry	Substrate Concentration (mmol/mL)	Conversion (%) [b]	Yield (%) [b]
1	0.5	42.3	42.3
2	0.4	46.0	46.0
3	0.3	46.4	46.4
4	0.2	51.6	51.6

[a] Reaction conditions: catalyst methanesulfonic acid (0.025 mmol, 2.4 mg), 30% H_2O_2 (1.15 equ.), solvent (2.0 mL H_2O), 25 °C, 24.0 h; [b] Conversion and selectivity were determined by GC with *p*-xylene as internal standard, product was determined by comparison with the authentic sample.

Table S7. Effect of catalyst loading on the oxidation of methyl phenyl sulfide with H_2O_2 catalyzed by *p*-toluenesulfonic acid [a].

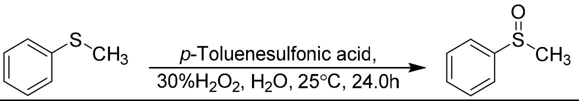

Entry	Catalyst Loading (%)	Conversion (%) [b]	Yield (%) [b]
1	0.0	39.1	39.1
2	2.5	45.8	45.8
3	5.0	47.8	47.8
4	7.5	57.2	56.9
5	10.0	64.4	64.1
6	12.5	75.5	75.1
7	15.0	82.5	82.0
8	17.5	88.2	87.6
9	20.0	96.1	95.5
10	22.5	99.4	98.6
**11**	**25.0**	**99.7**	**98.8**

[a] Reaction conditions: substrate (1.0 mmol, 124.2 mg), 30% H_2O_2 (1.15 mmol, 130 μL), solvent (2.0 mL H_2O), 25 °C, 24.0 h; [b] Conversion and selectivity were determined by GC with *p*-xylene as internal standard, product was determined by comparison with the authentic sample.

Table S8. Effect of the substrate amount on the oxidation of methyl phenyl sulfide with H_2O_2 catalyzed by *p*-toluenesulfonic acid [a].

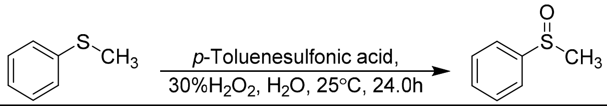

Entry	Substrate Concentration (mmol/mL)	Conversion (%) [b]	Yield (%) [b]
1	0.5	99.7	98.8
2	1.0	99.8	98.5
3	2.0	99.9	98.1
4	3.0	99.9	97.9
5	4.0	99.9	98.1
6	5.0	99.9	98.1

[a] Reaction conditions: catalyst *p*-toluenesulfonic acid (0.25 mmol, 43.1 mg), 30% H_2O_2 (1.15 equ.), solvent (2.0 mL H_2O), 25 °C, 24.0 h; [b] Conversion and selectivity were determined by GC with *p*-xylene as internal standard, product was determined by comparison with the authentic sample.

Table S9. Effect of the substrate amount on the oxidation of methyl phenyl sulfide with H_2O_2 catalyzed by *p*-toluenesulfonic acid [a].

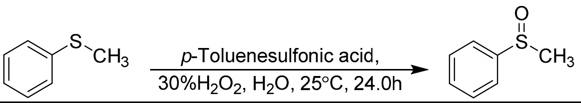

Entry	Substrate Concentration (mmol/mL)	Conversion (%) [b]	Yield (%) [b]
1	0.5	45.9	45.9
2	0.4	44.4	44.4
3	0.3	51.9	51.9
4	0.2	53.4	53.4

[a] Reaction conditions: catalyst *p*-toluenesulfonic acid (0.025 mmol, 4.3 mg), 30% H_2O_2 (1.15 equ.), solvent (2.0 mL H_2O), 25 °C, 24.0 h. [b] Conversion and selectivity were determined by GC with *p*-xylene as internal standard, product was determined by comparison with the authentic sample.

Table S10. Effect of catalyst loading on the oxidation of methyl phenyl sulfide with H_2O_2 catalyzed by methanoic acid [a].

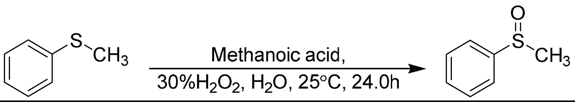

Entry	Catalyst Loading (%)	Conversion (%) [b]	Yield (%) [b]
1	0.0	39.1	39.1
2	100.0	58.7	58.5
3	150.0	63.4	63.2
4	200.0	79.3	78.9
5	250.0	85.8	85.4
6	300.0	89.8	89.4
7	350.0	97.3	96.8
**8**	**400.0**	**99.1**	**98.6**
9	450.0	99.3	97.4
10	500.0	99.3	95.9

[a] Reaction conditions: substrate (1.0 mmol, 124.2 mg), 30% H_2O_2 (1.15 mmol, 130 μL), solvent (2.0 mL H_2O), 25 °C, 24.0 h; [b] Conversion and selectivity were determined by GC with *p*-xylene as internal standard, product was determined by comparison with the authentic sample.

Table S11. Effect of the substrate amount on the oxidation of methyl phenyl sulfide with H_2O_2 catalyzed by methanoic acid [a].

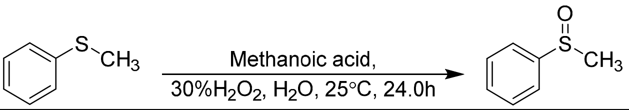

Entry	Substrate Concentration (mmol/mL)	Conversion (%) [b]	Yield (%) [b]
1	0.50	99.4	98.1
2	0.75	99.6	98.0
3	1.00	99.6	98.3
4	1.25	99.7	98.3
5	1.50	99.7	98.1
6	1.75	99.8	97.9
7	2.00	99.8	98.5

[a] Reaction conditions: catalyst methanoic acid (4.00 mmol, 184.1 mg), 30% H_2O_2 (1.15 equ.), solvent (2.0 mL H_2O), 25 °C, 24.0 h; [b] Conversion and selectivity were determined by GC with *p*-xylene as internal standard, product was determined by comparison with the authentic sample.

Table S12. Effect of the substrate amount on the oxidation of methyl phenyl sulfide with H2O2 catalyzed by methanoic acid [a].

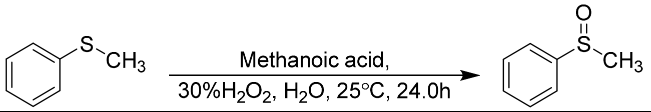

Entry	Substrate Concentration (mmol/mL)	Conversion (%) [b]	Yield (%) [b]
1	0.5	41.9	41.9
2	0.4	39.4	39.4
3	0.3	46.9	46.9
4	0.2	48.4	48.4

[a] Reaction conditions: catalyst methanoic acid (1.00 mmol, 46.0 mg), 30% H_2O_2 (1.15 equ.), solvent (2.0 mL H_2O), 25 °C, 24.0 h; [b] Conversion and selectivity were determined by GC with *p*-xylene as internal standard, product was determined by comparison with the authentic sample.

Table S13. Effect of catalyst loading on the oxidation of methyl phenyl sulfide with H2O2 catalyzed by acetic acid [a].

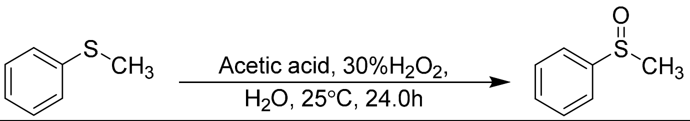

Entry	Catalyst Loading (%)	Conversion (%) [b]	Yield (%) [b]
1	0.0	41.5	41.5
2	100.0	54.9	54.7
3	150.0	61.9	61.8
4	200.0	66.8	66.7
5	250.0	70.8	70.7
6	300.0	75.2	75.0
7	350.0	86.8	86.6
8	400.0	93.8	93.6
9	450.0	95.3	95.1
10	500.0	96.6	96.4
**11**	**550.0**	**99.1**	**98.9**
12	600.0	99.1	98.9

[a] Reaction conditions: substrate (1.0 mmol, 124.2 mg), 30% H_2O_2 (1.15 mmol, 130 μL), solvent (2.0 mL H_2O), 25 °C, 24.0 h; [b] Conversion and selectivity were determined by GC with *p*-xylene as internal standard, product was determined by comparison with the authentic sample.

Table S14. Effect of the substrate amount on the oxidation of methyl phenyl sulfide with H_2O_2 catalyzed by acetic acid [a].

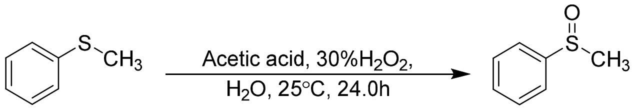

Entry	Substrate Concentration (mmol/mL)	Conversion (%) [b]	Yield (%) [b]
1	0.50	99.2	98.9
2	0.75	98.6	98.3
3	1.00	98.5	98.1
4	1.25	99.3	98.9
5	1.50	99.5	99.0
6	1.75	99.7	99.1
7	2.00	99.6	99.0
8	2.25	99.6	98.8
9	2.50	99.1	98.5
10	2.75	98.9	98.1
11	3.00	99.1	97.8

[a] Reaction conditions: catalyst acetic acid (5.50 mmol, 330.0 mg), 30% H_2O_2 (1.15 equ.), solvent (2.0 mL H_2O), 25 °C, 24.0 h; [b] Conversion and selectivity were determined by GC with *p*-xylene as internal standard, product was determined by comparison with the authentic sample.

Table S15. Effect of the substrate amount on the oxidation of methyl phenyl sulfide with H_2O_2 catalyzed by acetic acid [a].

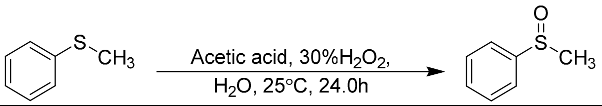

Entry	Substrate Concentration (mmol/mL)	Conversion (%) [b]	Yield (%) [b]
1	0.5	46.8	46.8
2	0.4	47.2	47.2
3	0.3	47.6	47.6
4	0.2	58.1	58.1

[a] Reaction conditions: catalyst acetic acid (1.00 mmol, 60.1 mg), 30% H_2O_2 (1.15 equ.), solvent (2.0 mL H_2O), 25 °C, 24.0 h; [b] Conversion and selectivity were determined by GC with *p*-xylene as internal standard, product was determined by comparison with the authentic sample.

Table S16. Effect of pH value on the oxidation of methyl phenyl sulfide with H_2O_2 catalyzed by methanesulfonic acid [a].

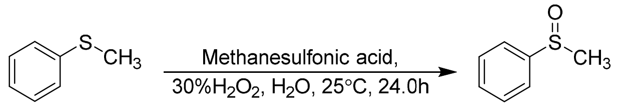

Entry	pH Value	Conversion (%) [b]	Yield (%) [b]
1	2.00	43.7	43.6
2	1.90	49.8	49.6
3	1.80	57.9	57.6
4	1.70	67.5	67.2
5	1.60	72.7	72.3
6	1.50	78.2	77.4
7	1.40	98.0	97.2
**8**	**1.30**	**99.8**	**98.7**
9	1.20	99.7	98.4
10	1.10	99.5	97.9
11	1.00	99.3	96.4

[a] Reaction conditions: substrate (1.0 mmol, 124.2 mg), 30% H_2O_2 (1.15 mmol, 130 μL), catalyst and solvent (2.0 mL aqueous solution of methanesulfonic acid), 25 °C, 24.0 h; [b] Conversion and selectivity were determined by GC with *p*-xylene as internal standard, product was determined by comparison with the authentic sample.

Table S17. Effect of pH value on the oxidation of methyl phenyl sulfide with H_2O_2 catalyzed by *p*-toluenesulfonic acid [a].

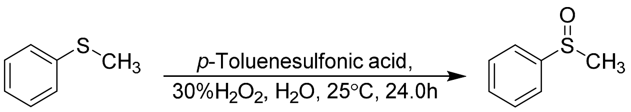

Entry	pH Value	Conversion (%) [b]	Yield (%) [b]
1	2.00	53.7	53.5
2	1.90	55.8	55.6
3	1.80	57.5	57.2
4	1.70	67.0	66.6
5	1.60	75.5	75.0
6	1.50	85.6	85.0
7	1.40	96.8	96.0
**8**	**1.30**	**99.7**	**98.5**
9	1.20	99.8	98.2
10	1.10	99.6	96.8
11	1.00	99.5	93.9

[a] Reaction conditions: substrate (1.0 mmol, 124.2 mg), 30% H_2O_2 (1.15 mmol, 130 μL), catalyst and solvent (2.0 mL aqueous solution of *p*-toluenesulfonic acid), 25 °C, 24.0 h; [b] Conversion and selectivity were determined by GC with *p*-xylene as internal standard, product was determined by comparison with the authentic sample.

Table S18. Effect of pH value on the oxidation of methyl phenyl sulfide with H_2O_2 catalyzed by methanoic acid [a].

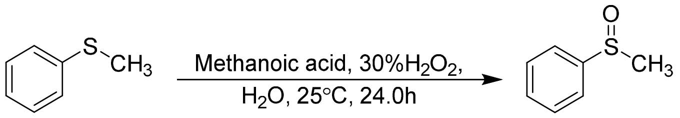

Entry	pH Value	Conversion (%) [b]	Yield (%) [b]
1	2.00	49.3	49.1
2	1.90	50.3	50.2
3	1.80	53.8	53.3
4	1.70	57.1	56.6
5	1.60	71.3	70.8
6	1.50	74.9	74.3
7	1.40	98.4	97.9
**8**	**1.30**	**99.6**	**99.1**
9	1.20	99.6	98.7
10	1.10	99.8	98.1
11	1.00	99.2	97.3

[a] Reaction conditions: substrate (1.0 mmol, 124.2 mg), 30% H_2O_2 (1.15 mmol, 130 μL), catalyst and solvent (2.0 mL aqueous solution of methanoic acid), 25 °C, 24.0 h; [b] Conversion and selectivity were determined by GC with *p*-xylene as internal standard, product was determined by comparison with the authentic sample.

Table S19. Effect of pH value on the oxidation of methyl phenyl sulfide with H_2O_2 catalyzed by sulfuric acid [a].

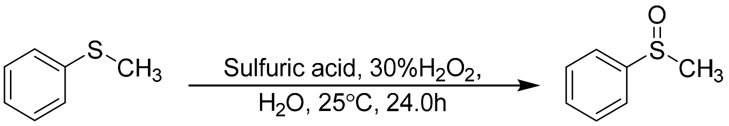

Entry	pH Value	Conversion (%) [b]	Yield (%) [b]
1	2.00	52.5	52.2
2	1.90	53.5	53.3
3	1.80	56.9	56.7
4	1.70	59.1	58.8
5	1.60	73.3	72.8
6	1.50	76.1	75.6
7	1.40	98.5	97.9
**8**	**1.30**	**99.7**	**98.6**
9	1.20	99.6	97.8
10	1.10	99.7	98.2
11	1.00	99.2	97.1

[a] Reaction conditions: substrate (1.0 mmol, 124.2 mg), 30% H_2O_2 (1.15 mmol, 130 μL), catalyst and solvent (2.0 mL aqueous solution of sulfuric acid), 25 °C, 24.0 h; [b] Conversion and selectivity were determined by GC with *p*-xylene as internal standard, product was determined by comparison with the authentic sample.

Table S20. Effect of pH value on the oxidation of methyl phenyl sulfide with H_2O_2 catalyzed by hydrochloric acid [a].

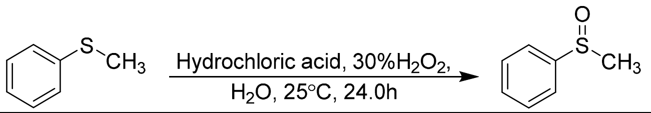

Entry	pH Value	Conversion (%) [b]	Yield (%) [b]
1	2.00	48.1	47.9
2	1.90	52.8	52.6
3	1.80	61.5	61.1
4	1.70	64.5	64.1
5	1.60	68.3	68.0
6	1.50	88.9	88.0
7	1.40	99.2	98.3
**8**	**1.30**	**99.7**	**98.8**
9	1.20	99.6	98.4
10	1.10	99.3	97.1
11	1.00	99.3	97.3

[a] Reaction conditions: substrate (1.0 mmol, 124.2 mg), 30% H_2O_2 (1.15 mmol, 130 μL), catalyst and solvent (2.0 mL aqueous solution of hydrochloric acid), 25 °C, 24.0 h; [b] Conversion and selectivity were determined by GC with *p*-xylene as internal standard, product was determined by comparison with the authentic sample.

Table S21. Effect of pH value on the oxidation of methyl phenyl sulfide with H_2O_2 catalyzed by trifluoroacetic acid [a].

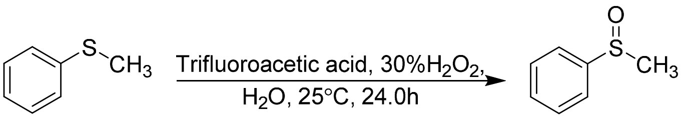

Entry	pH Value	Conversion (%) [b]	Yield (%) [b]
1	2.00	52.9	52.7
2	1.90	60.8	60.6
3	1.80	66.1	65.8
4	1.70	66.2	65.9
5	1.60	78.0	77.5
6	1.50	89.0	88.2
7	1.40	97.7	96.6
**8**	**1.30**	**98.8**	**97.7**
9	1.20	99.0	96.4
10	1.10	95.1	92.0
11	1.00	95.1	85.3

[a] Reaction conditions: substrate (1.0 mmol, 124.2 mg), 30% H_2O_2 (1.15 mmol, 130 μL), catalyst and solvent (2.0 mL aqueous solution of trifluoroacetic acid), 25 °C, 24.0 h; [b] Conversion and selectivity were determined by GC with *p*-xylene as internal standard, product was determined by comparison with the authentic sample.

2. ^{1}H-NMR Spectra of Product Sulfoxides

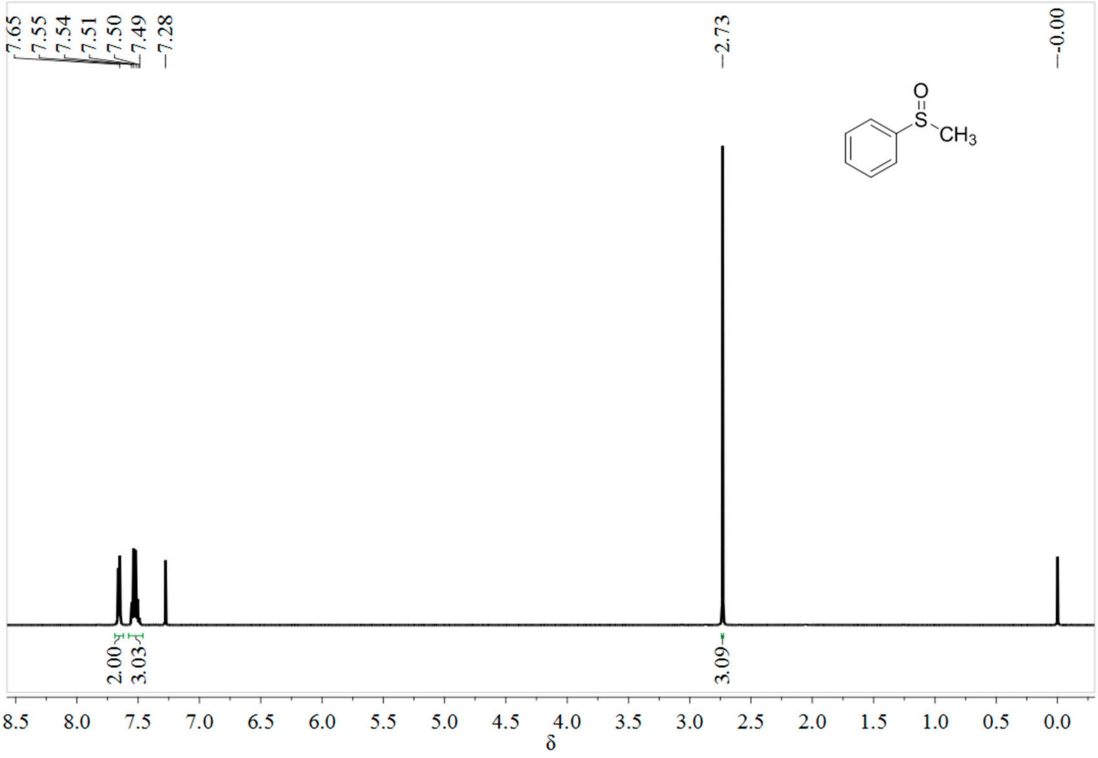

Figure S1. ^{1}H-NMR spectrum of methyl phenyl sulfoxide.

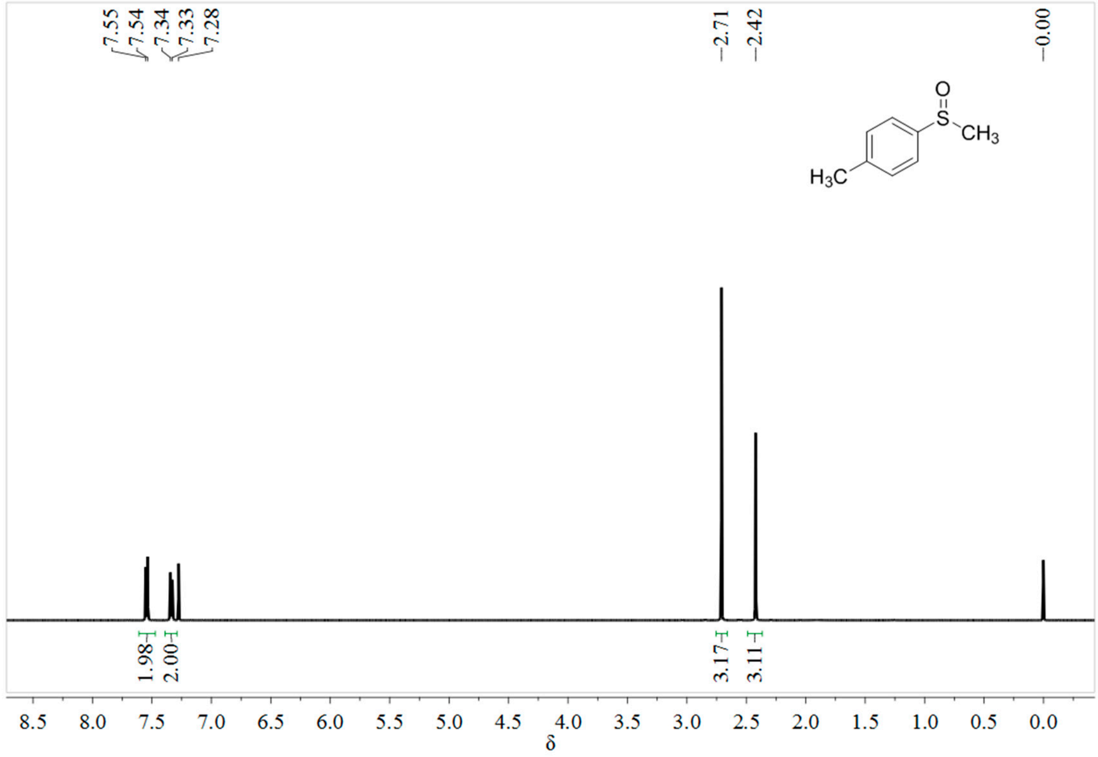

Figure S2. ^{1}H-NMR spectrum of methyl (4-methylphenyl) sulfoxide.

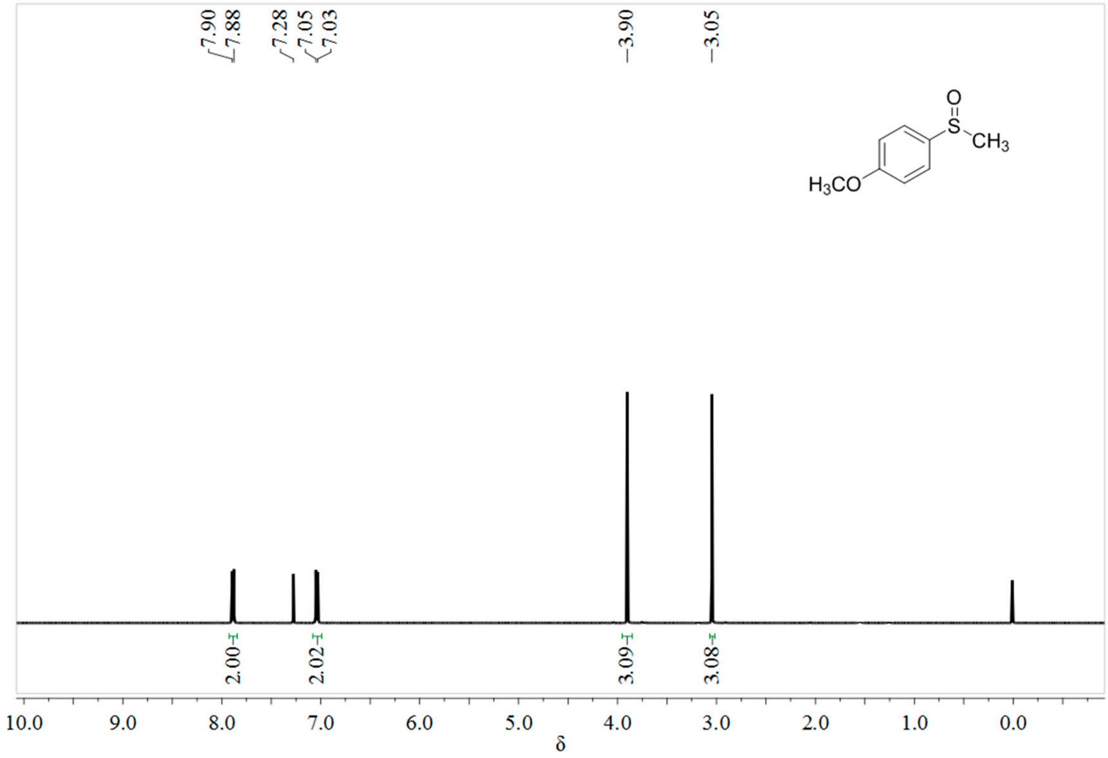

Figure S3. ^{1}H-NMR spectrum of methyl (4-methoxylphenyl) sulfoxide.

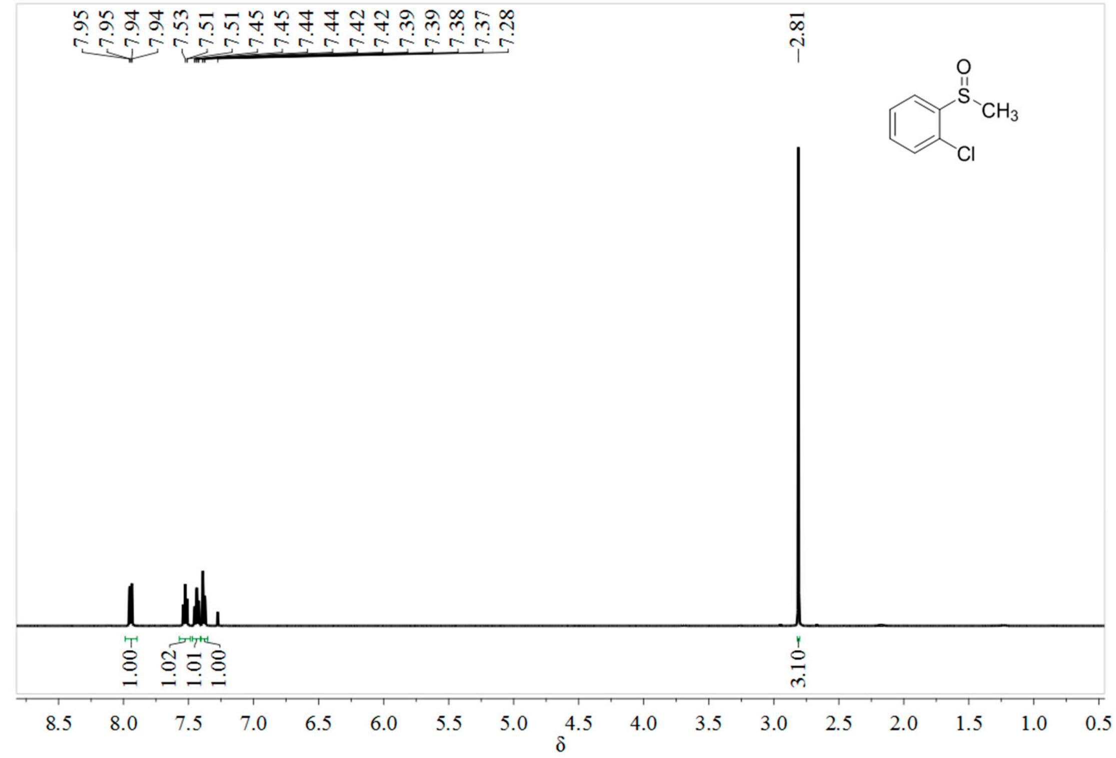

Figure S4. ^{1}H-NMR spectrum of methyl (2-chlorophenyl) sulfoxide.

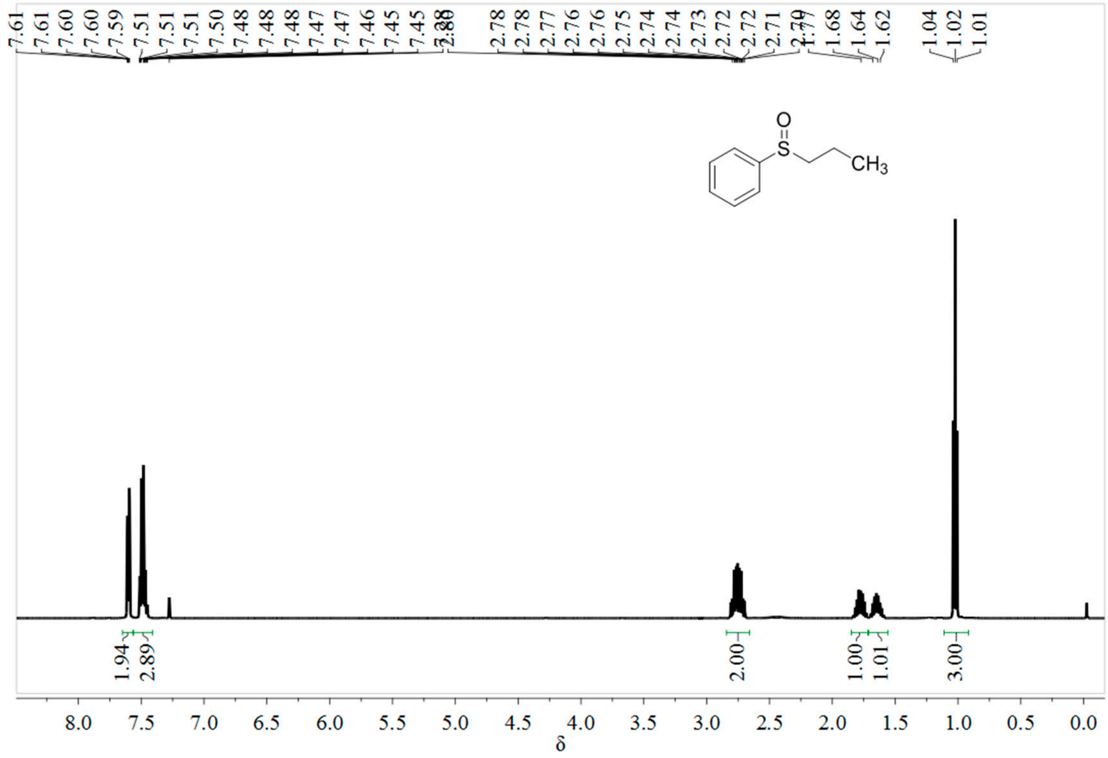

Figure S5. ^{1}H-NMR spectrum of *n*-propyl phenyl sulfoxide.

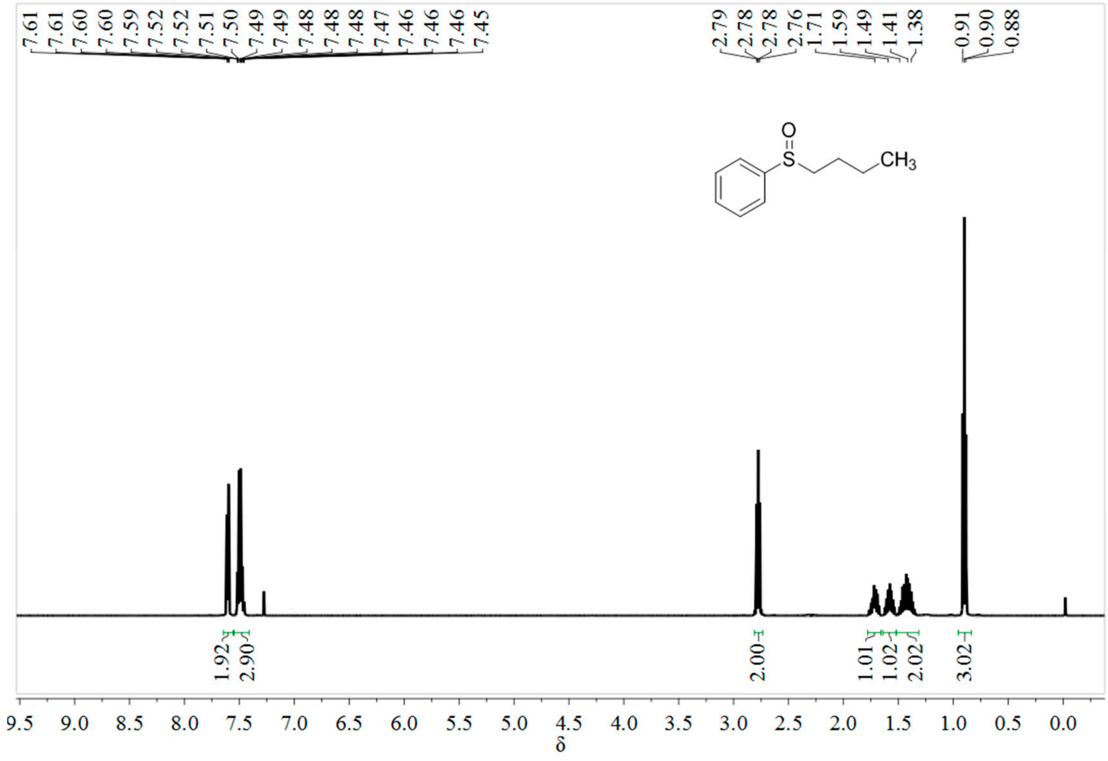

Figure S6. ^{1}H-NMR spectrum of *n*-butyl phenyl sulfoxide.

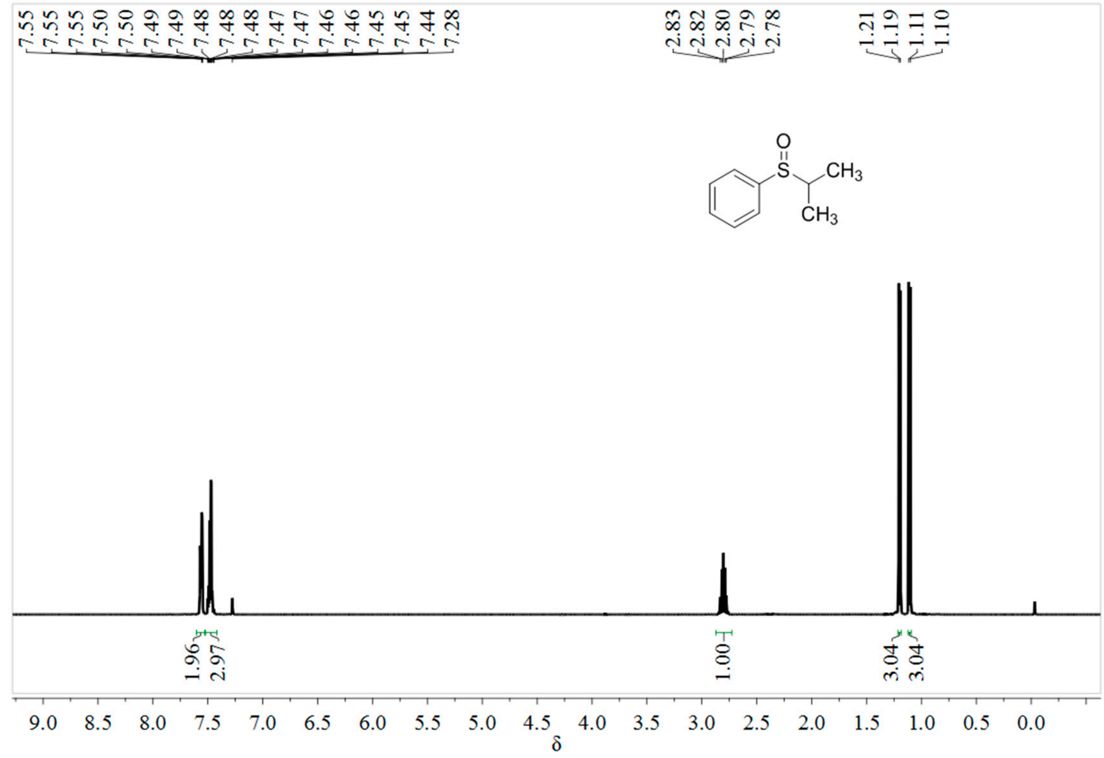

Figure S7. ^{1}H-NMR spectrum of isopropyl phenyl sulfoxide.

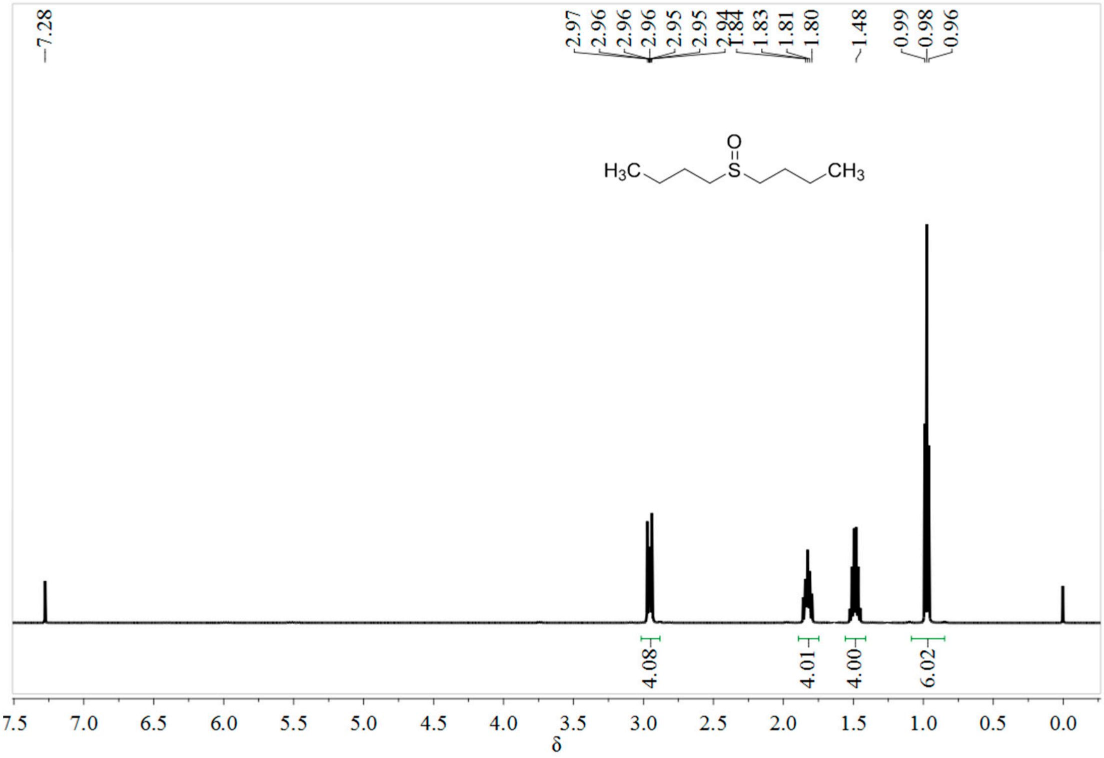

Figure S8. ^{1}H-NMR spectrum of *n*-butyl sulfoxide.

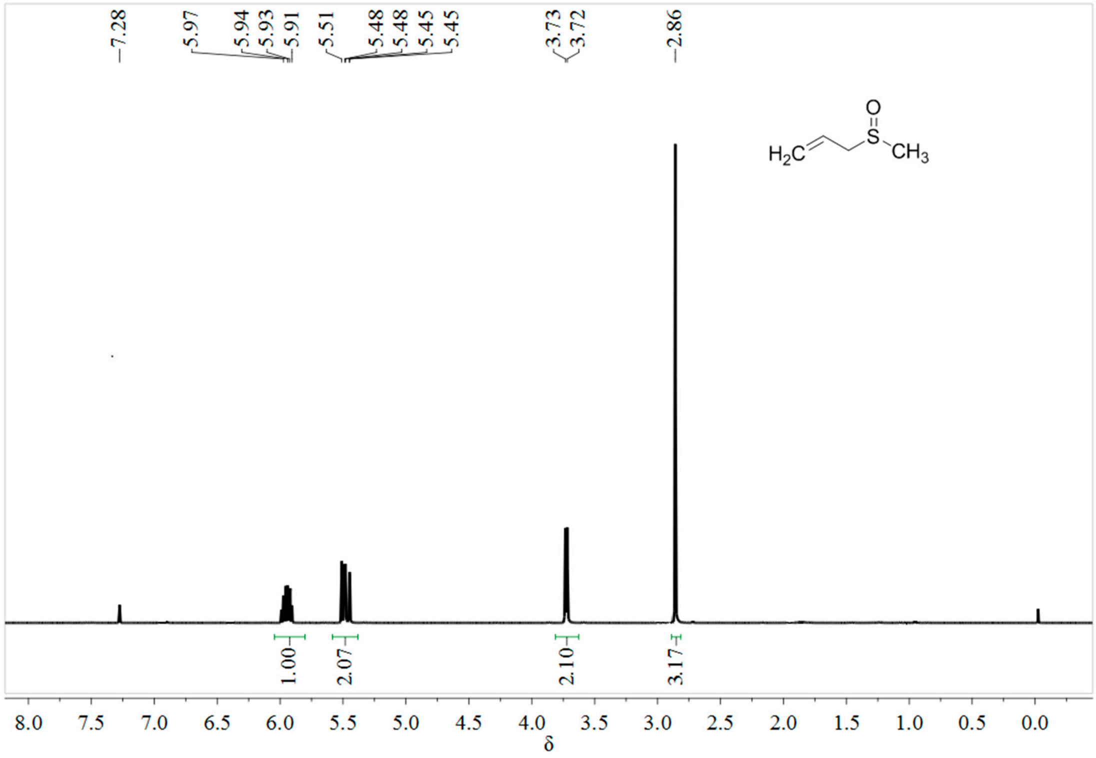

Figure S9. ^{1}H-NMR spectrum of allyl methyl sulfoxide.

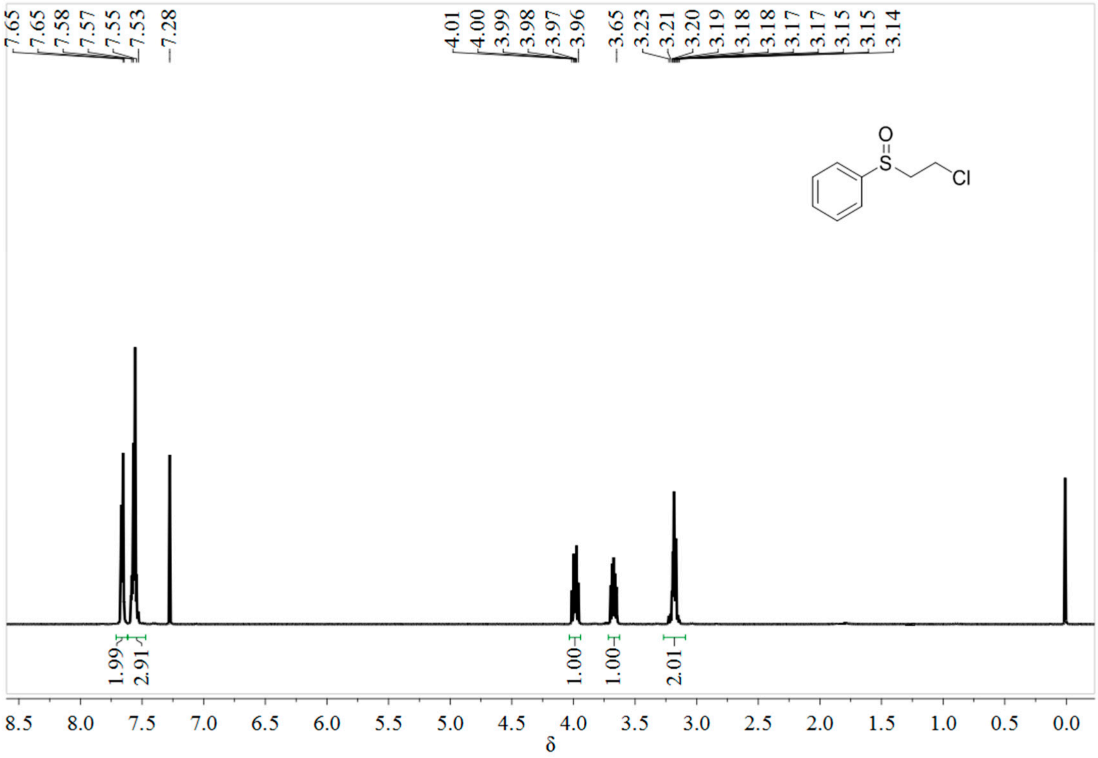

Figure S10. ^{1}H-NMR spectrum of 2-chloroethyl phenyl sulfoxide.

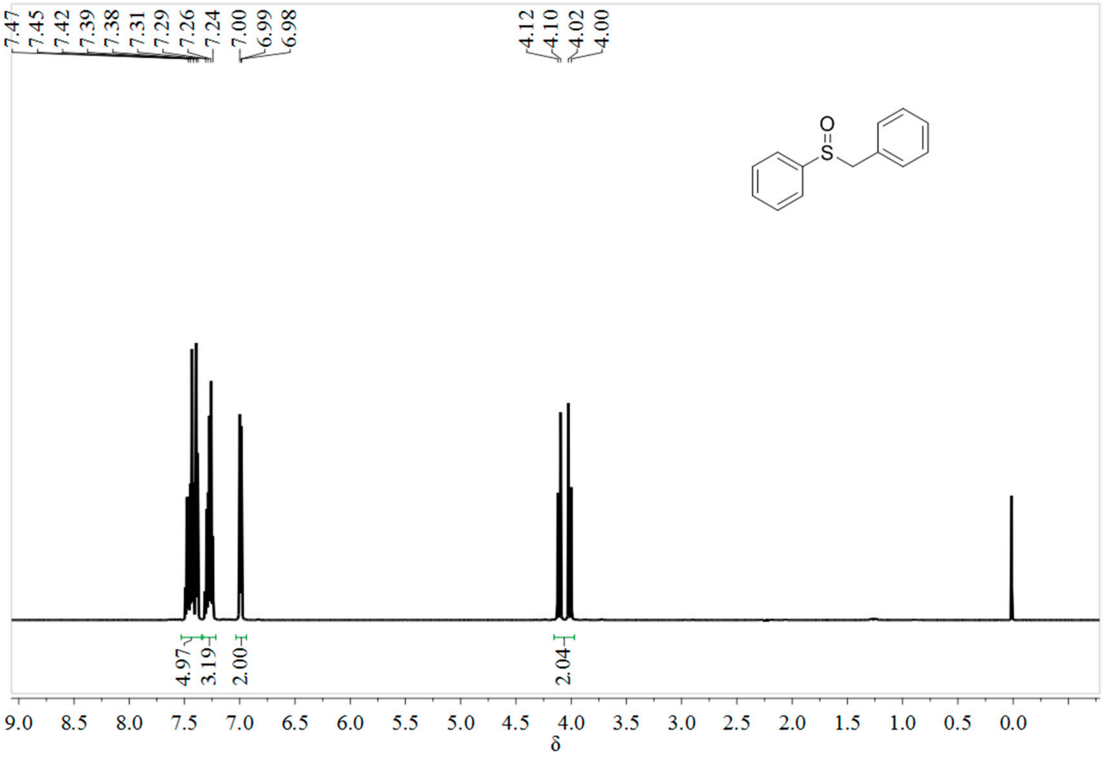

Figure S11. ^{1}H-NMR spectrum of benzyl phenyl sulfoxide.

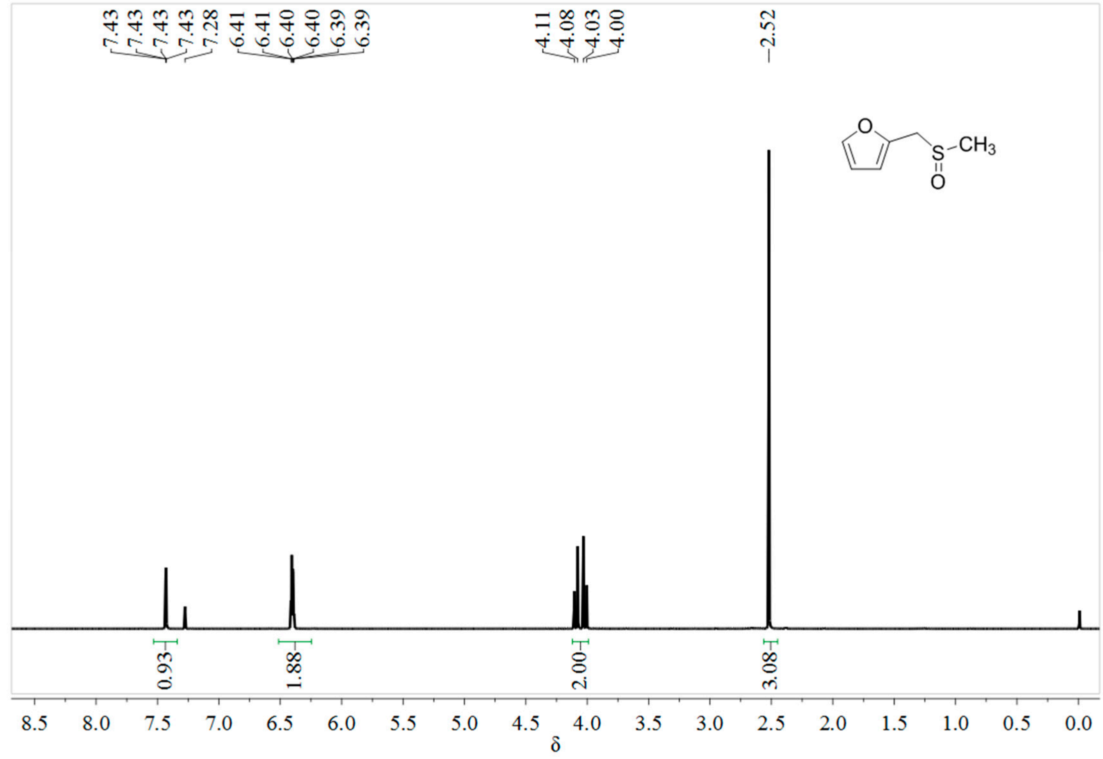

Figure S12. ^{1}H-NMR spectrum of furfuryl methyl sulfoxide.

3. ^{13}C-NMR Spectra of Product Sulfoxides

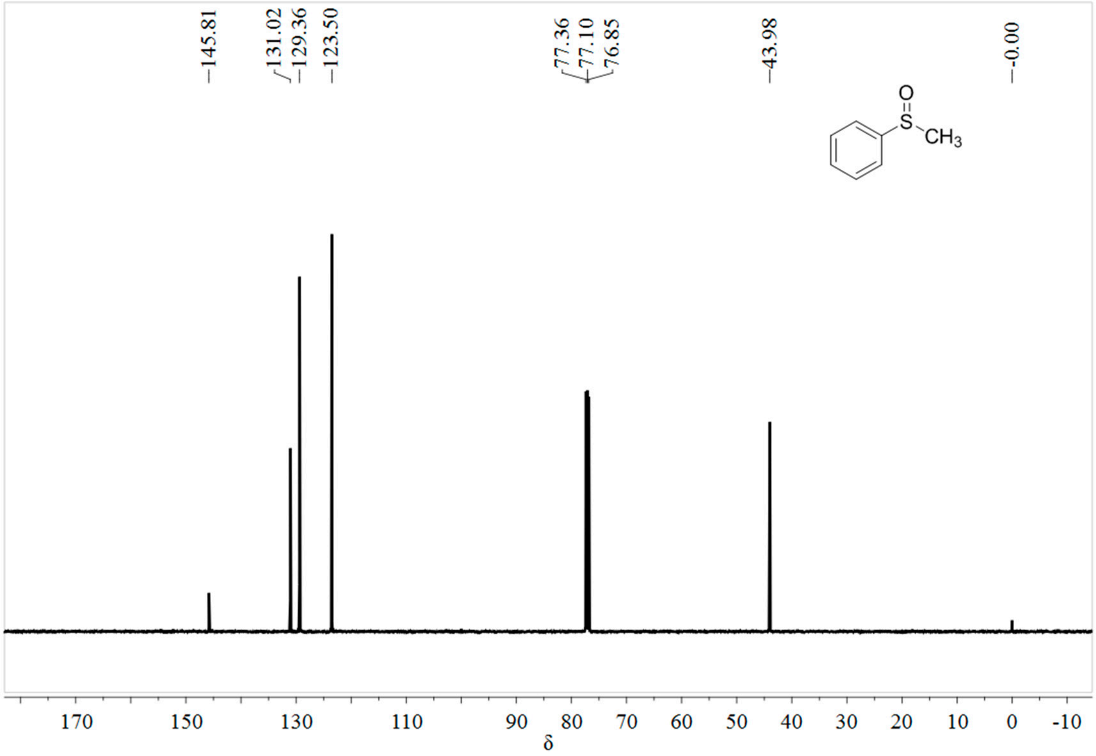

Figure S13. ^{13}C-NMR spectrum of methyl phenyl sulfoxide.

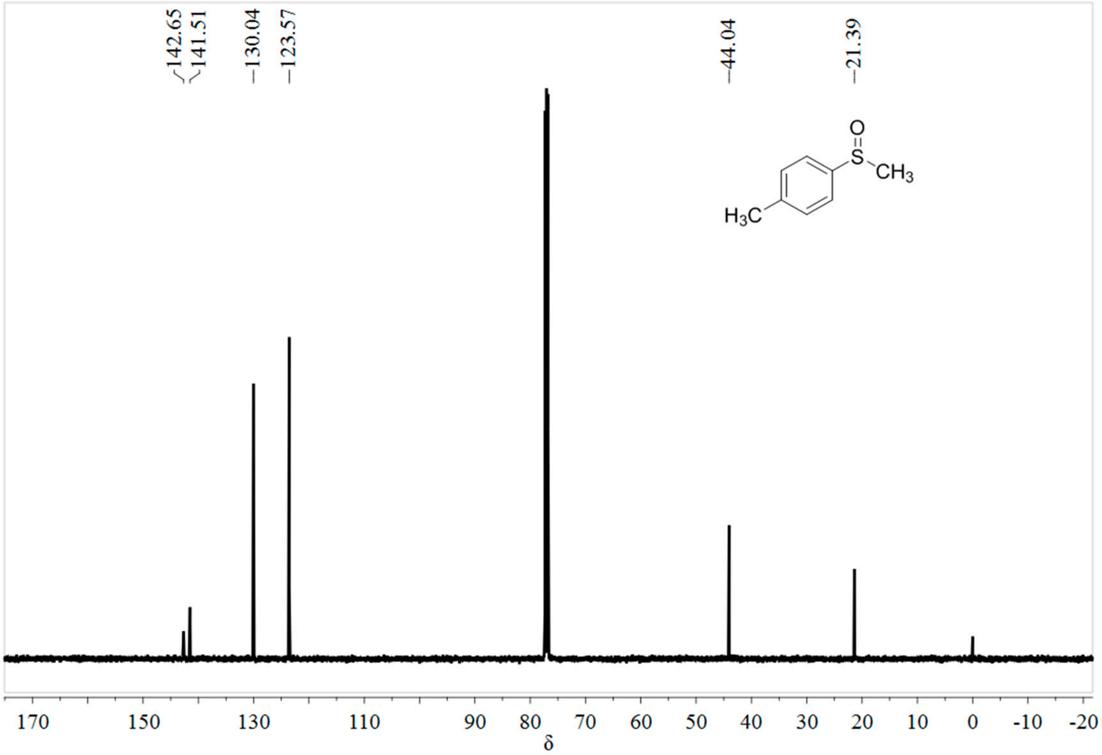

Figure S14. ^{13}C-NMR spectrum of methyl (4-methylphenyl) sulfoxide.

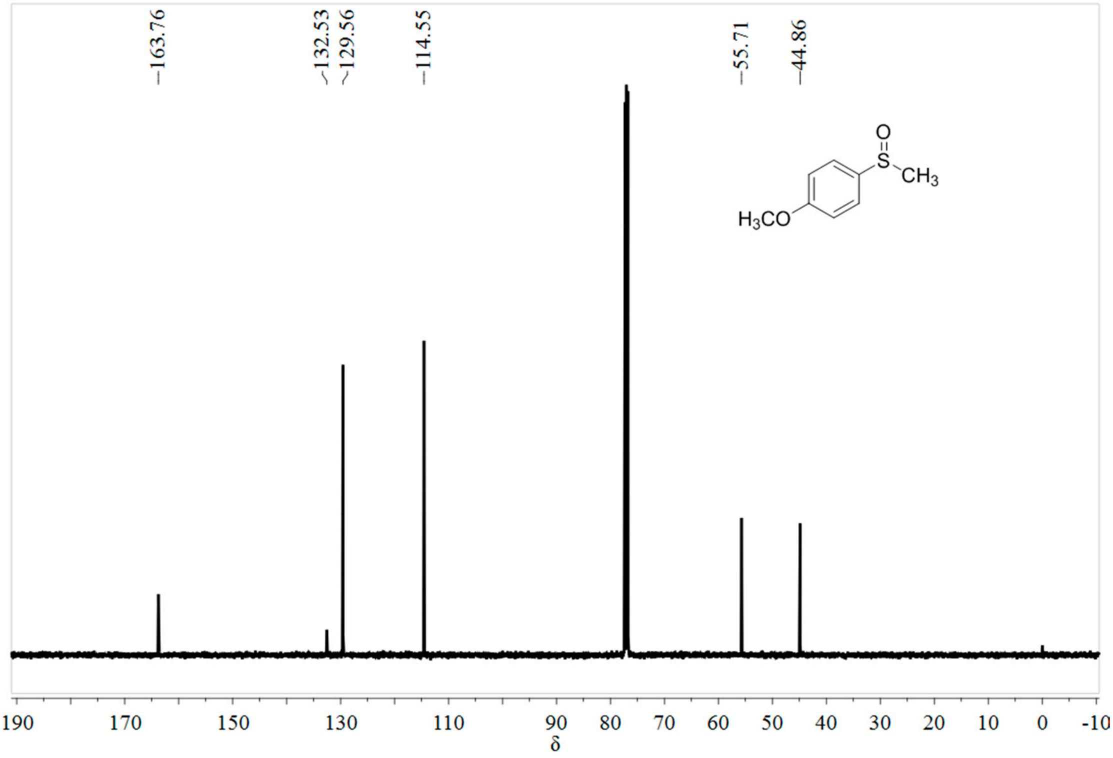

Figure S15. ^{13}C-NMR spectrum of methyl (4-methoxylphenyl) sulfoxide.

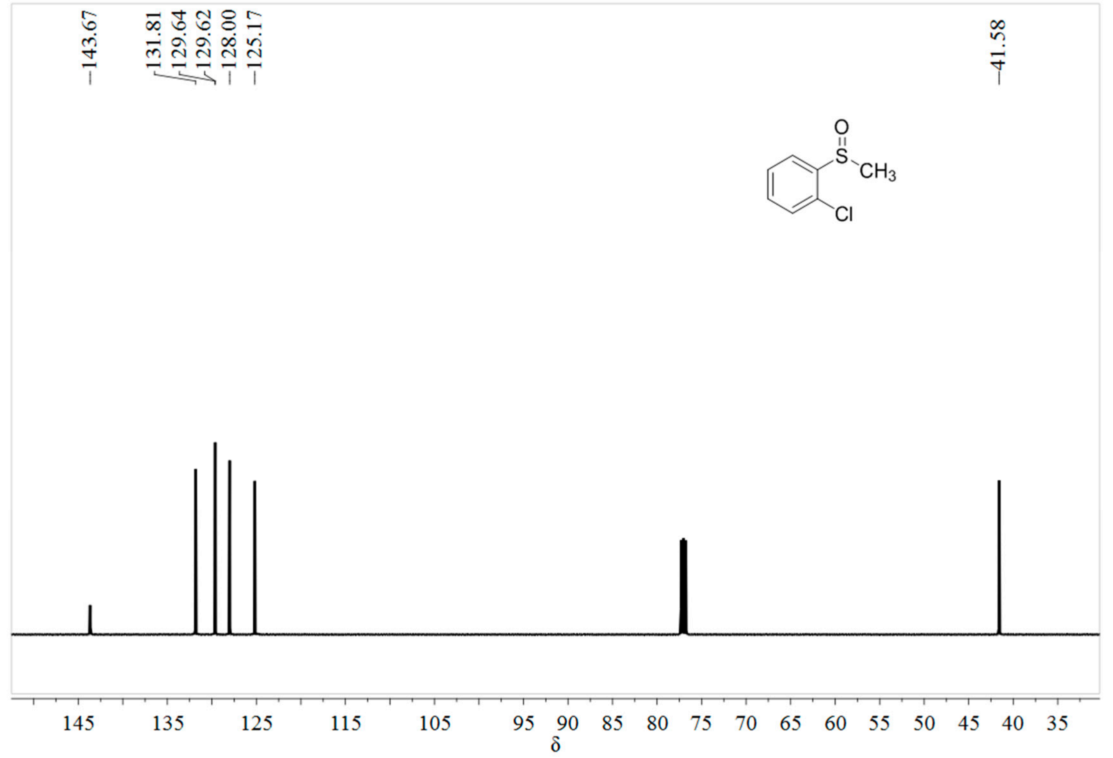

Figure S16. ^{13}C-NMR spectrum of methyl (2-chlorophenyl) sulfoxide.

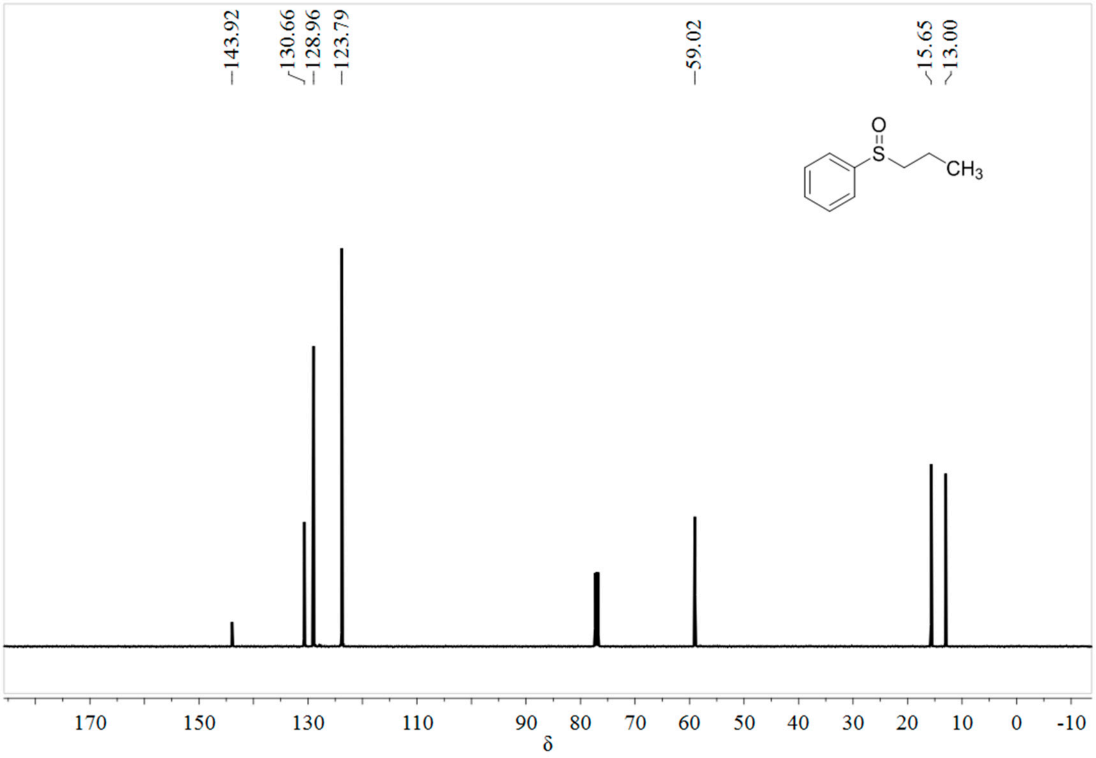

Figure S17. ^{13}C-NMR spectrum of *n*-propyl phenyl sulfoxide.

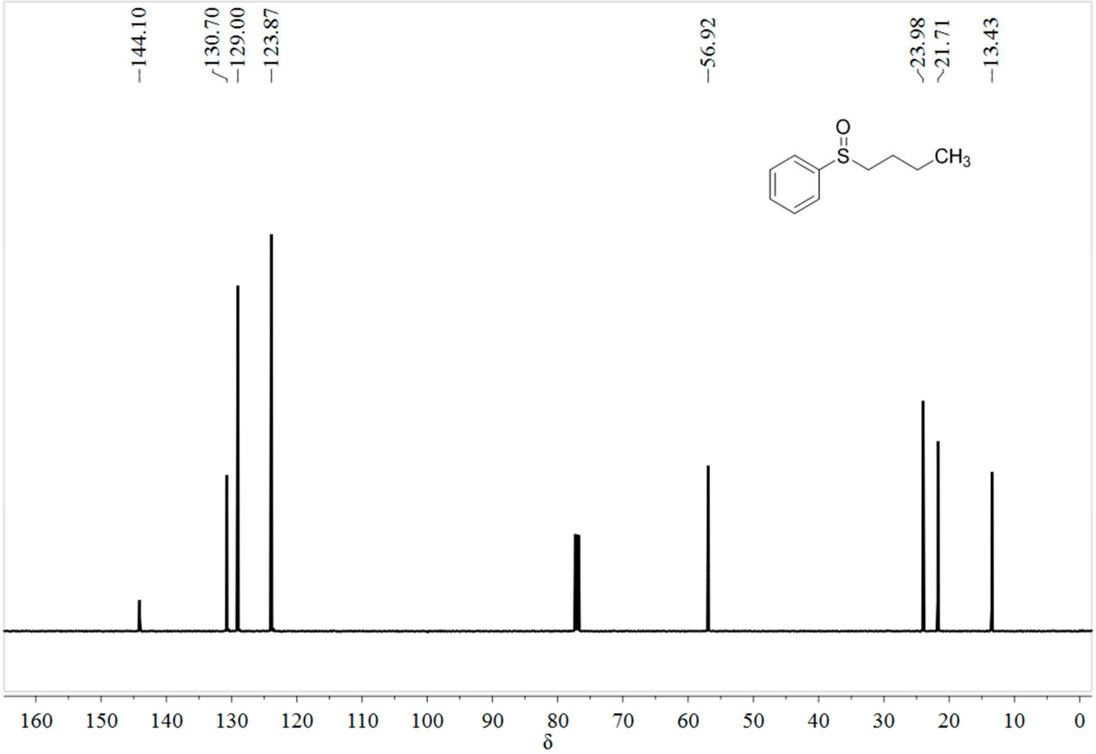

Figure S18. ^{13}C-NMR spectrum of *n*-butyl phenyl sulfoxide.

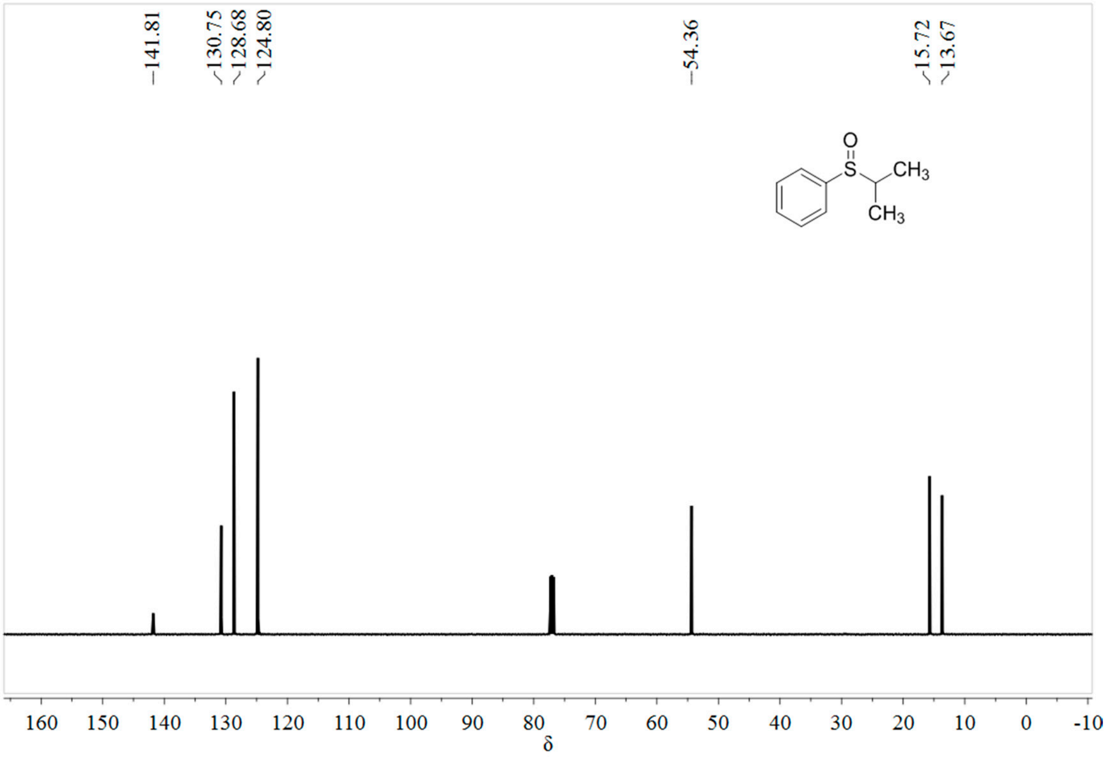

Figure S19. ^{13}C-NMR spectrum of isopropyl phenyl sulfoxide.

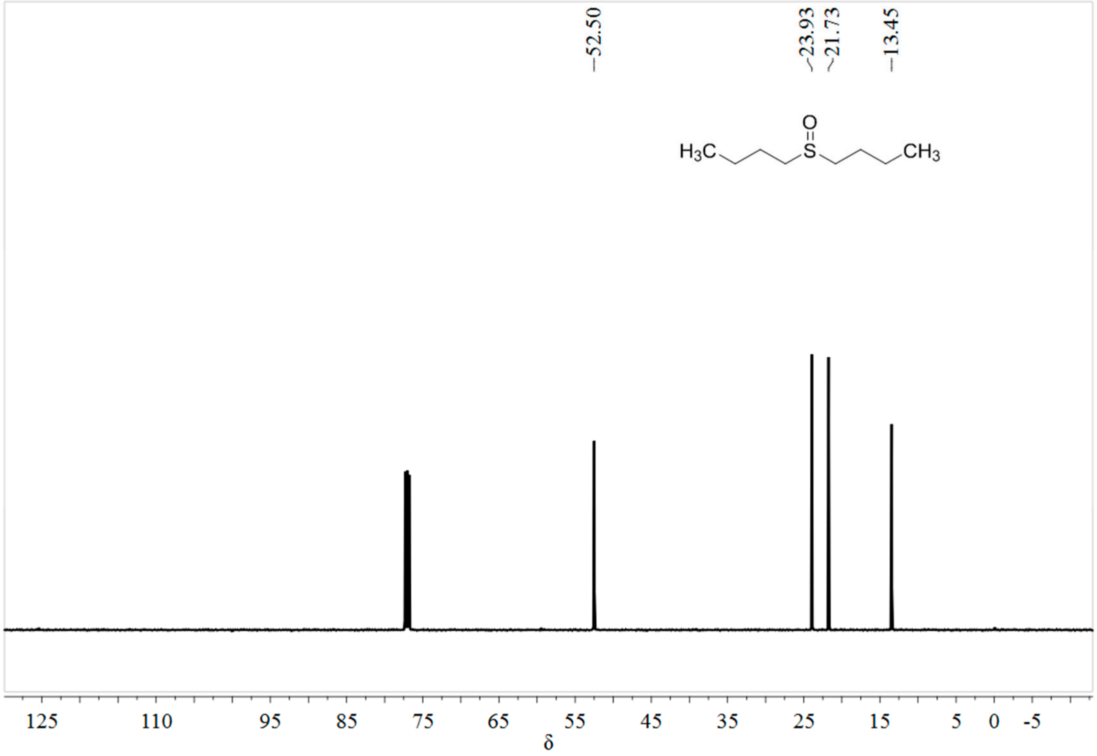

Figure S20. ^{13}C-NMR spectrum of *n*-butyl sulfoxide.

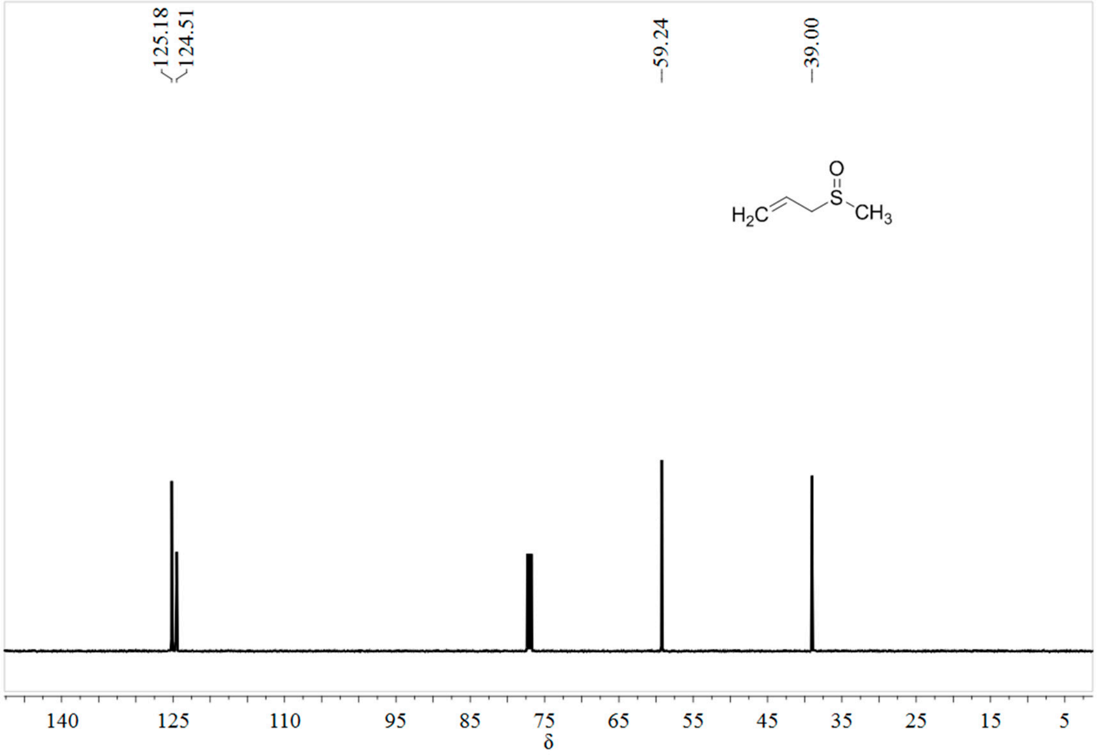

Figure S21. ^{13}C-NMR spectrum of allyl methyl sulfoxide.

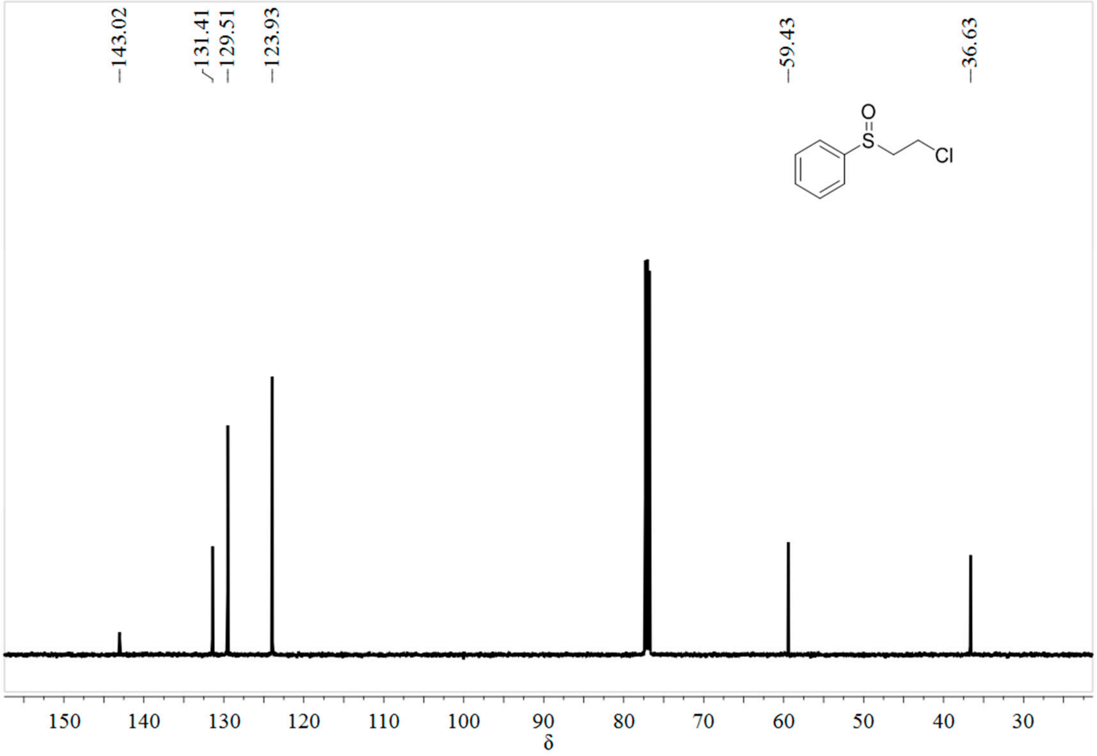

Figure S22. ^{13}C-NMR spectrum of 2-chloroethyl phenyl sulfoxide.

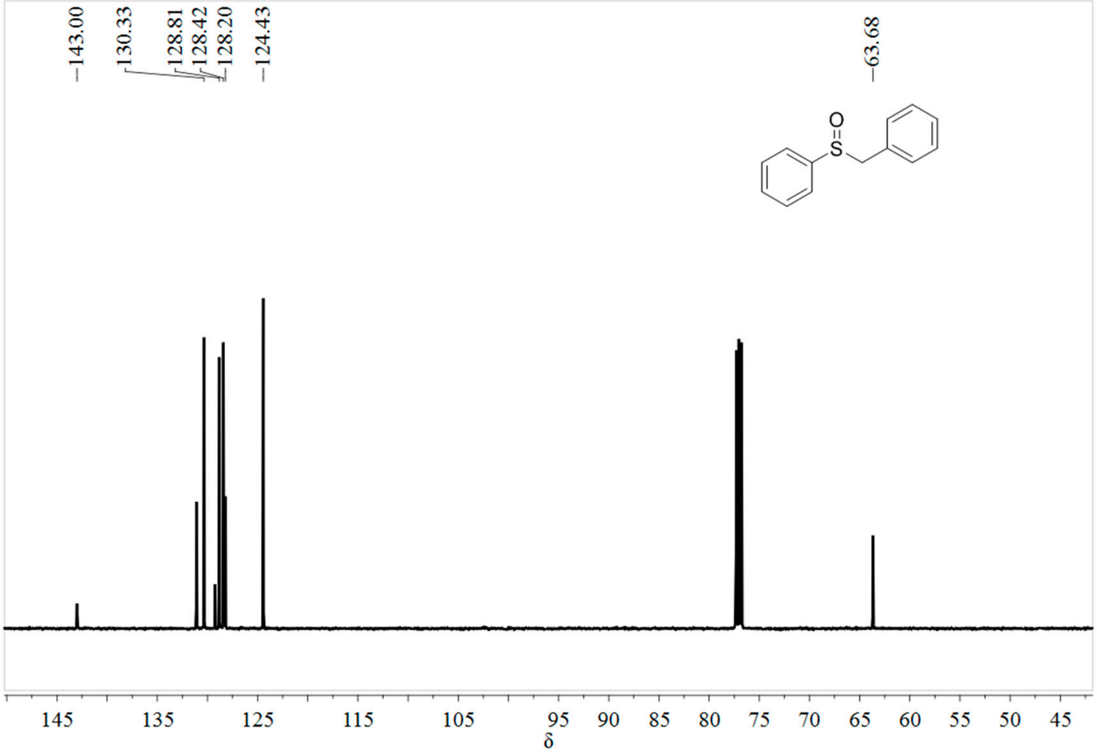

Figure S23. ^{13}C-NMR spectrum of benzyl phenyl sulfoxide.

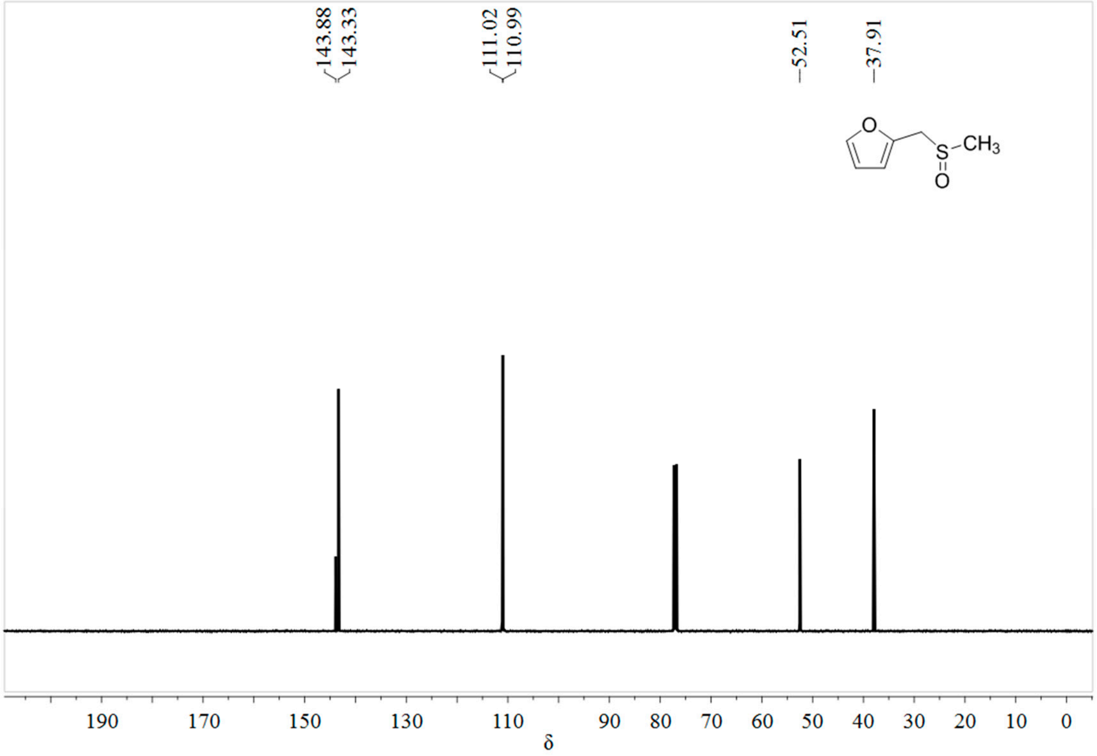

Figure S24. ^{13}C-NMR spectrum of furfuryl methyl sulfoxide.

4. NMR Data of Product Sulfoxides

Table S22. NMR Data of Product Sulfoxides.

No.	Molecule Structures	^{1}H- Data	^{13}C- Data
1	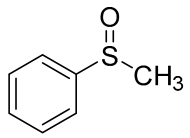	^{1}H-NMR (400 MHz, $CDCl_3$): δ = 7.65 (d, 2H), 7.54–7.49 (m, 3H), 2.73 ppm (s, 3H)	^{13}C-NMR (400 MHz, $CDCl_3$): δ = 145.81, 131.02, 129.36, 123.50, 43.98 ppm
2	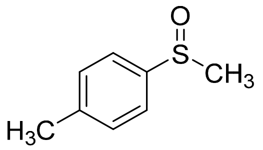	^{1}H-NMR (400MHz, $CDCl_3$): δ = 7.55 (d, 2H), 7.34 (d, 2H), 2.71 (s, 3H), 2.42 ppm (s, 3H)	^{13}C-NMR (400 MHz, $CDCl_3$): δ =142.65, 141.51, 130.04, 123.57, 44.04, 21.39 ppm
3	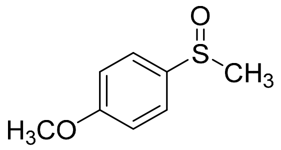	^{1}H-NMR (400 MHz, $CDCl_3$): δ = 7.90 (d, 2H), 7.05 (d, 2H), 3.90 (s, 3H), 3.05 ppm (s, 3H)	^{13}C-NMR (400 MHz, $CDCl_3$): δ = 163.76, 132.53, 129.56, 114.55, 55.71, 44.86 ppm
4	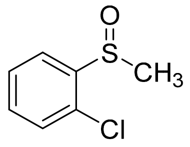	^{1}H-NMR (400 MHz, $CDCl_3$): δ = 7.95 (d, 1H), 7.51 (t, 1H), 7.45 (t, 1H), 7.39 (d, 1H), 2.81 ppm (s, 3H)	^{13}C-NMR (400 MHz, $CDCl_3$): δ = 143.67, 131.81, 129.64, 129.62, 128.00, 125.17, 41.58 ppm
5	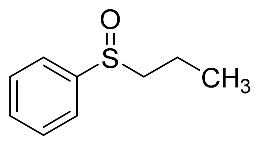	^{1}H-NMR (400 MHz, $CDCl_3$): δ = 7.61 (d, 2H), 7.51-7.45 (m, 3H), 2.78–2.70 (m, 2H), 1.77–1.62 (m, 2H), 1.02 ppm (t, 3H)	^{13}C-NMR (400 MHz, $CDCl_3$): δ =143.92, 130.66, 128.96, 123.79, 59.02, 15.65, 13.00 ppm
6	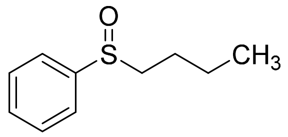	^{1}H-NMR (400 MHz, $CDCl_3$): δ = 7.61 (d, 2H), 7.52–7.45 (m, 3H), 2.78 (t, 2H), 1.76–1.68 (m, 1H), 1.62–1.54 (m, 1H), 1.50–1.34 (m, 2H), 0.90 ppm (t, 3H)	^{13}C-NMR (400 MHz, $CDCl_3$): δ =144.10, 130.70, 129.00, 123.87, 56.92, 23.98, 21.71, 13.43 ppm
7	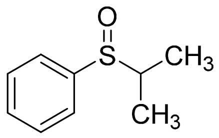	^{1}H-NMR (400 MHz, $CDCl_3$): δ = 7.55 (d, 2H), 7.50–7.44 (m, 3H), 2.83–2.78 (m, 1H), 1.21 (d, 3H), 1.11 ppm (d, 3H)	^{13}C-NMR (400 MHz, $CDCl_3$): δ =141.81, 130.75, 128.68, 124.80, 54.36, 15.72, 13.67 ppm
8	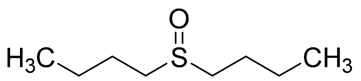	^{1}H-NMR (400 MHz, $CDCl_3$): δ = 2.97–2.94 (m, 4H), 1.86–1.80 (m, 4H), 1.52–1.45 (m, 4H), 0.98 ppm (t, 6H)	^{13}C-NMR (400 MHz, $CDCl_3$): δ =52.50, 23.93, 21.73, 13.45 ppm
9	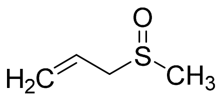	^{1}H-NMR (400 MHz, $CDCl_3$): δ = 5.97–5.91 (m, 1H), 5.51–5.45 (m, 2H), 3.73 (d, 2H), 2.86 ppm (s, 3H)	^{13}C-NMR (400 MHz, $CDCl_3$): δ =125.18, 124.51, 59.24, 39.00 ppm

Table S22. *Cont.*

No.	Molecule Structures	^{1}H- Data	^{13}C- Data
10	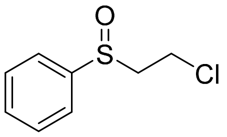	^{1}H-NMR (400 MHz, $CDCl_3$): δ = 7.67–7.65 (m, 2H), 7.59–7.53 (m, 3H), 4.01–3.96 (m, 1H), 3.70–3.65 (m, 1H), 3.23–3.14 ppm (m, 2H)	^{13}C-NMR (400 MHz, $CDCl_3$): δ =143.02, 131.41, 129.51, 123.93, 59.43, 36.63 ppm
11	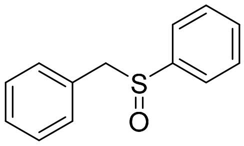	^{1}H-NMR (400 MHz, $CDCl_3$): δ = 7.49–7.38 (m, 5H), 7.32–7.24 (m, 3H), 7.00 (d, 2H), 4.12–4.00 ppm (m, 2H)	^{13}C-NMR (400 MHz, $CDCl_3$): δ =143.00, 131.09, 130.33, 129.24, 128.81, 128.42, 128.20, 124.43, 63.68 ppm
12	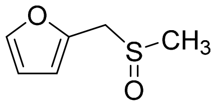	^{1}H-NMR (400 MHz, $CDCl_3$): δ = 7.43 (s, 1H), 6.41-6.39 (m, 2H), 4.11–4.00 (m, 2H), 2.52 ppm (s, 3H)	^{13}C-NMR (400 MHz, $CDCl_3$): δ =143.88, 143.33, 111.02, 110.99, 52.51, 37.91 ppm

5. ESI-MS Spectra of Product Sulfoxides

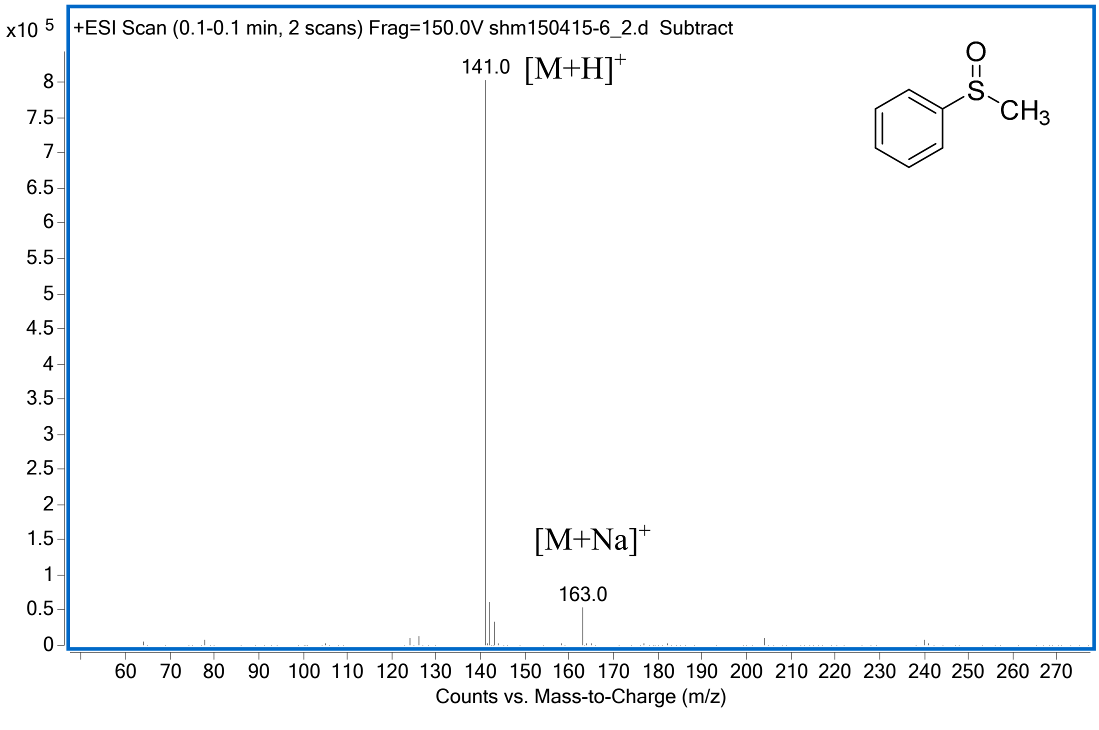

Figure S25. ESI-MS spectrum of methyl phenyl sulfoxide.

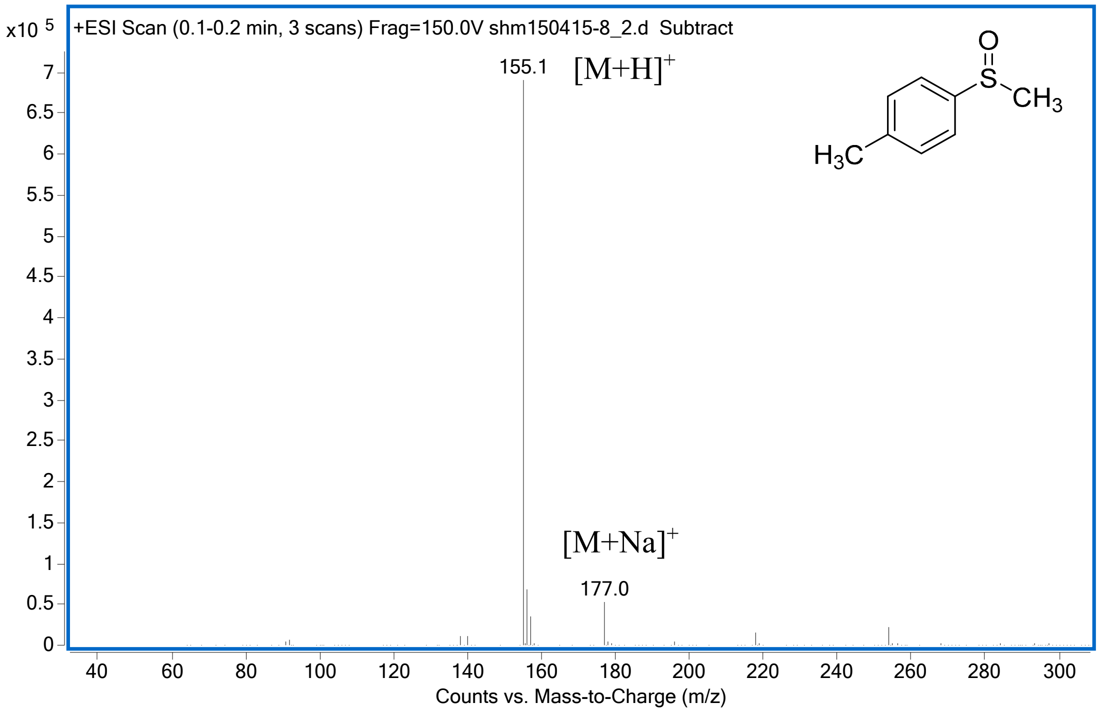

Figure S26. ESI-MS spectrum of methyl (4-methylphenyl) sulfoxide.

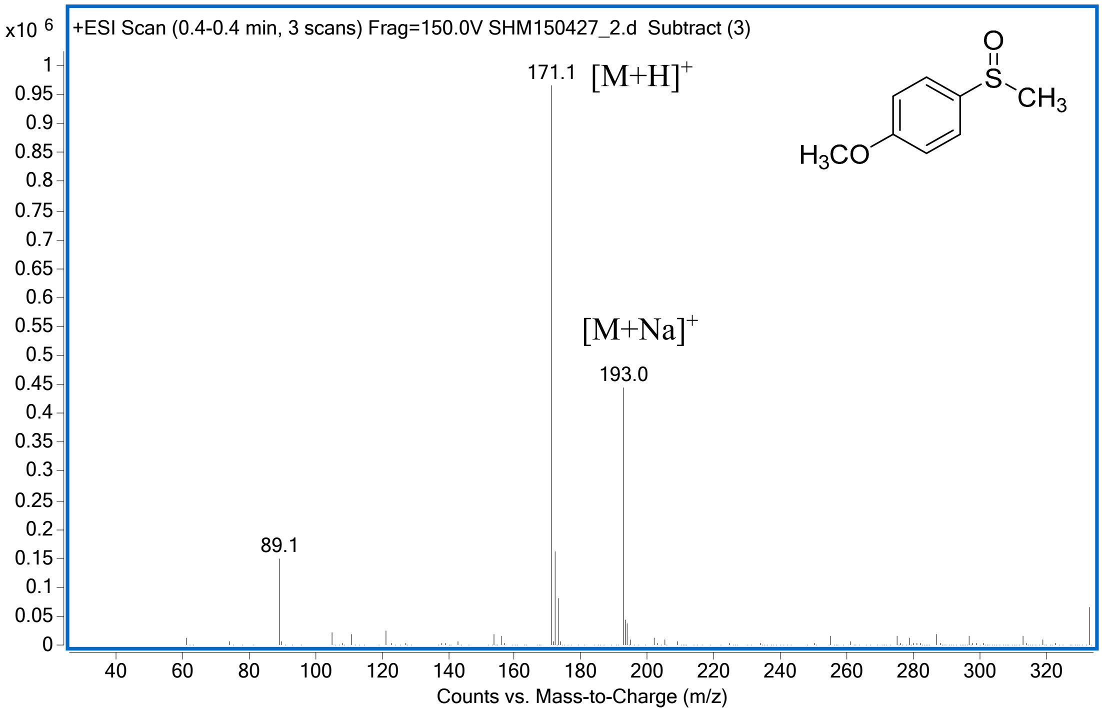

Figure S27. ESI-MS spectrum of methyl (4-methoxylphenyl) sulfoxide.

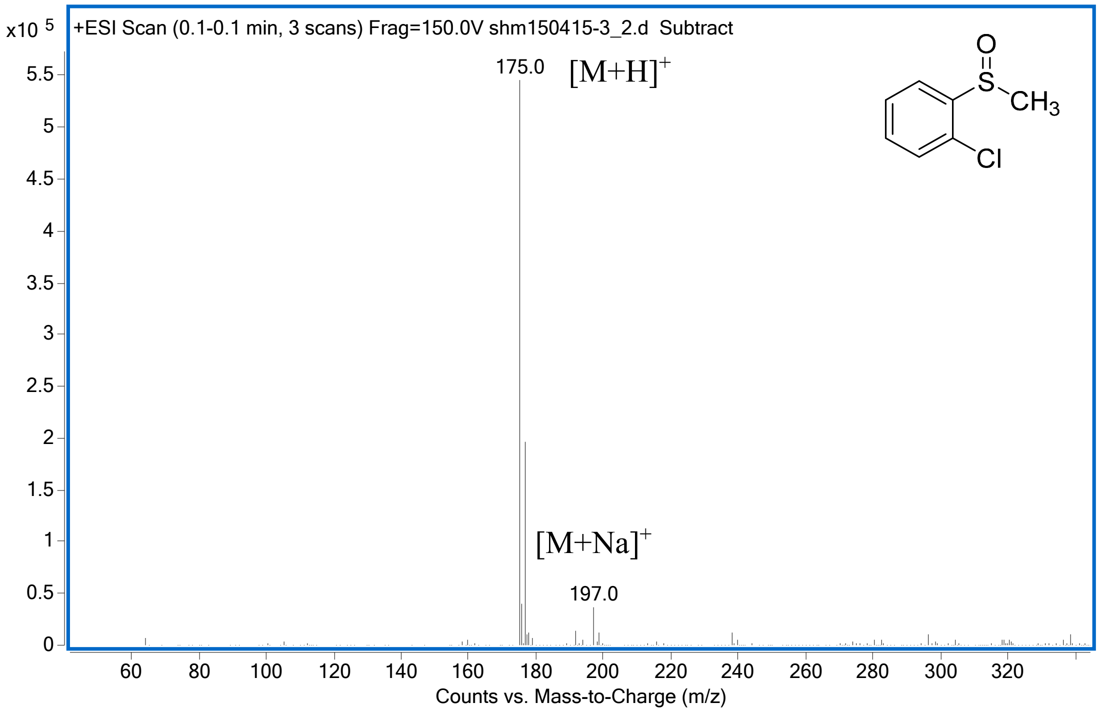

Figure S28. ESI-MS spectrum of methyl (2-chlorophenyl) sulfoxide.

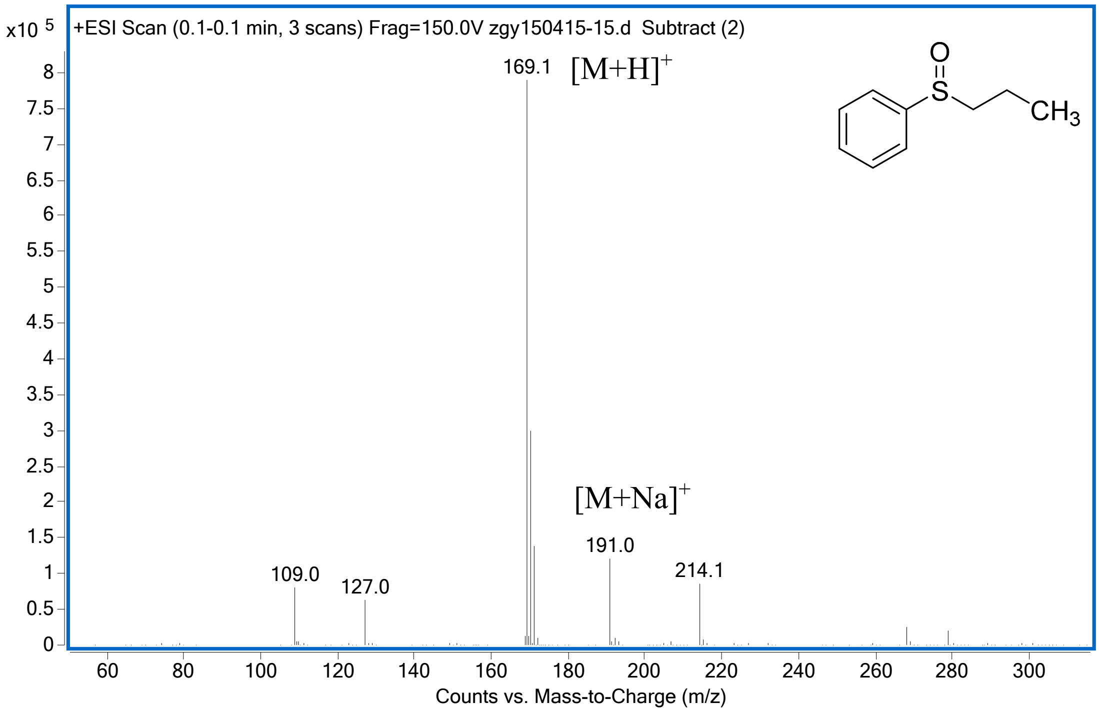

Figure S29. ESI-MS spectrum of *n*-propyl phenyl sulfoxide.

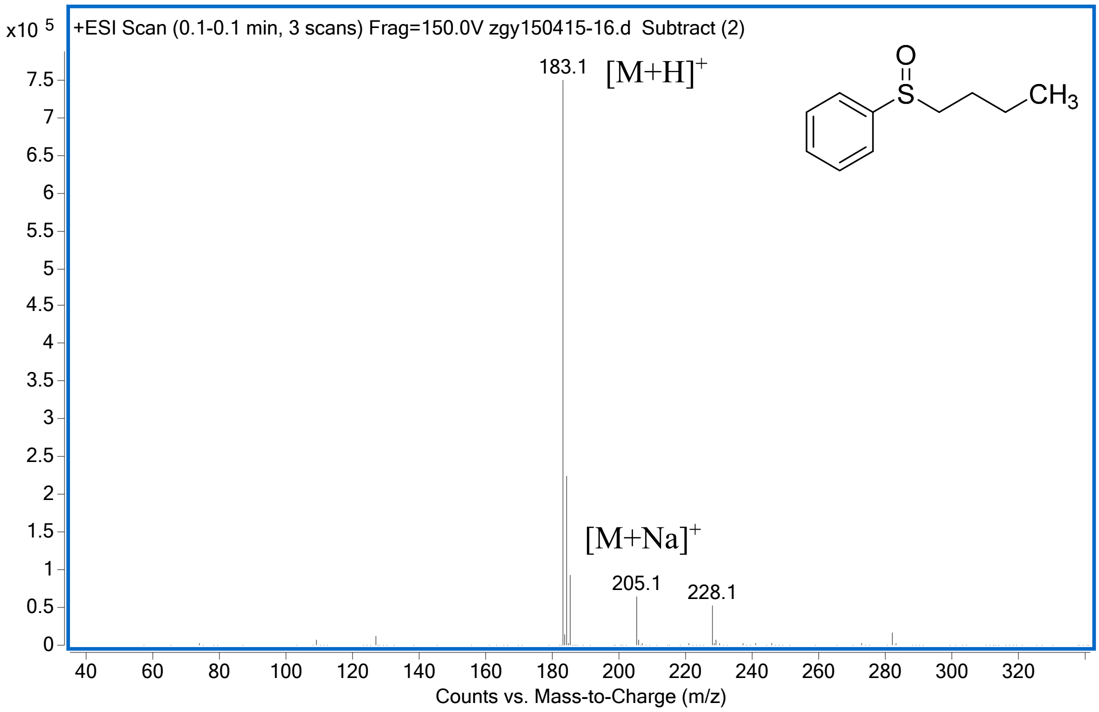

Figure S30. ESI-MS spectrum of *n*-butyl phenyl sulfoxide.

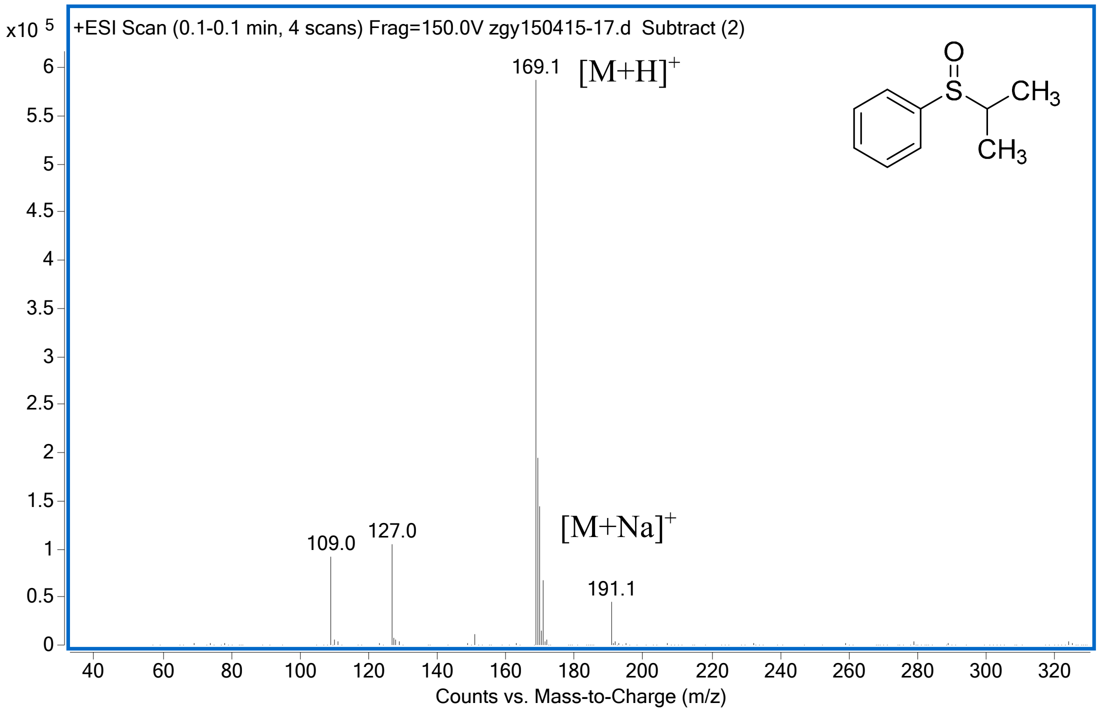

Figure S31. ESI-MS spectrum of isopropyl phenyl sulfoxide.

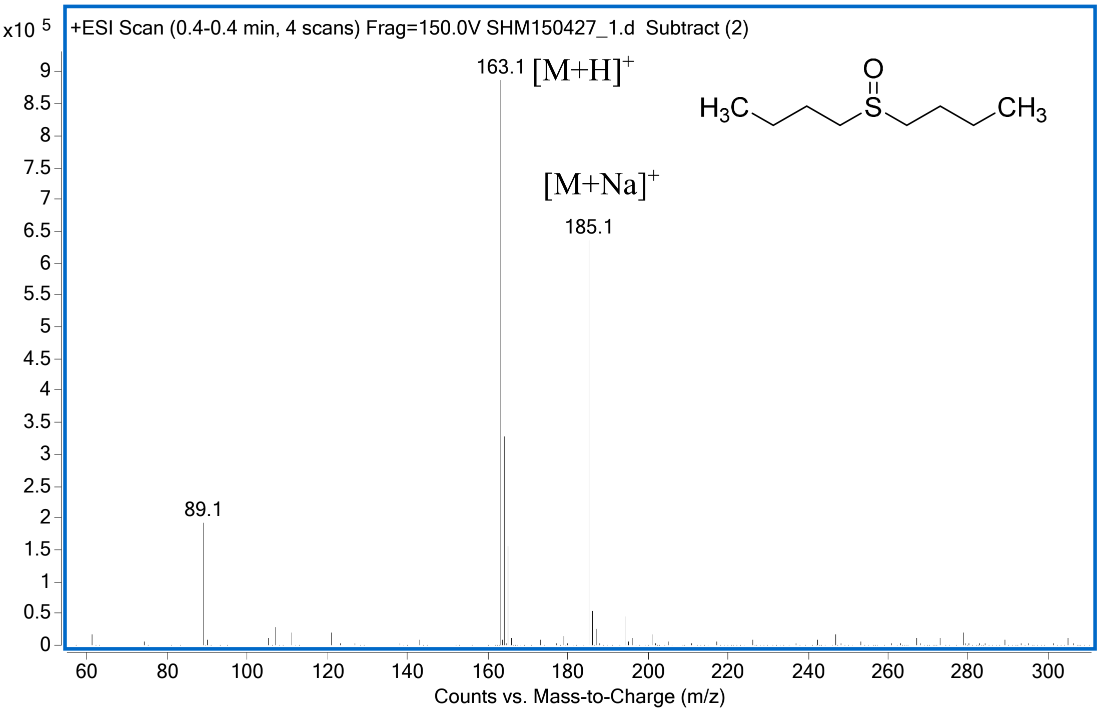

Figure S32. ESI-MS spectrum of *n*-butyl sulfoxide.

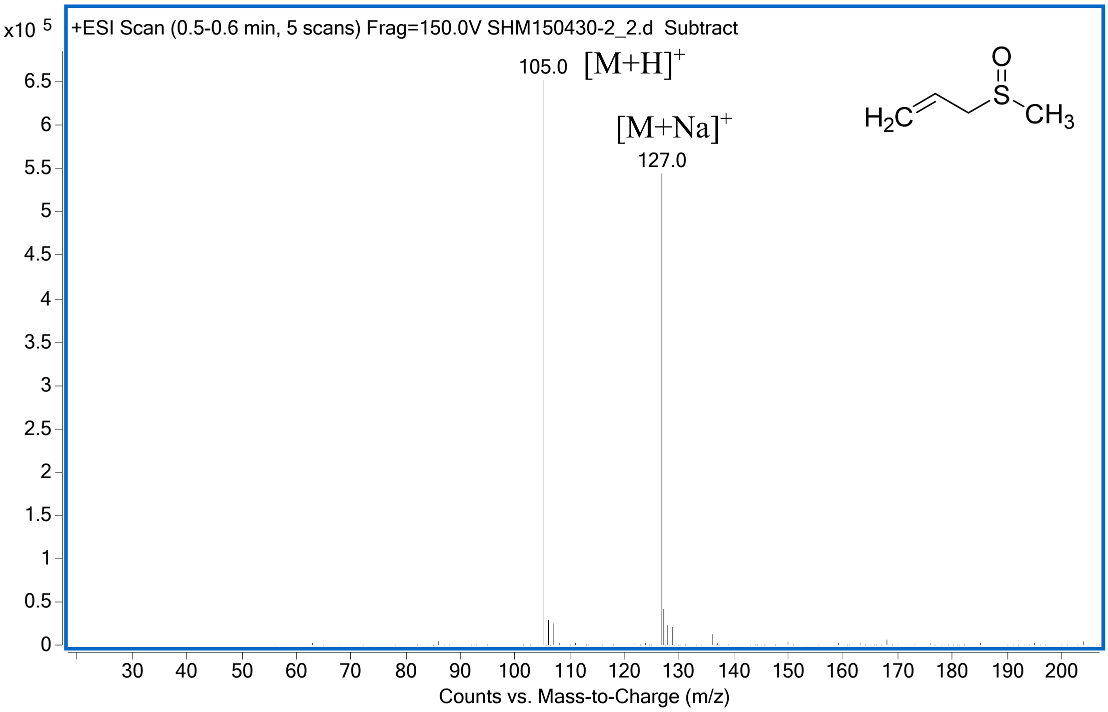

Figure S33. ESI-MS spectrum of allyl methyl sulfoxide.

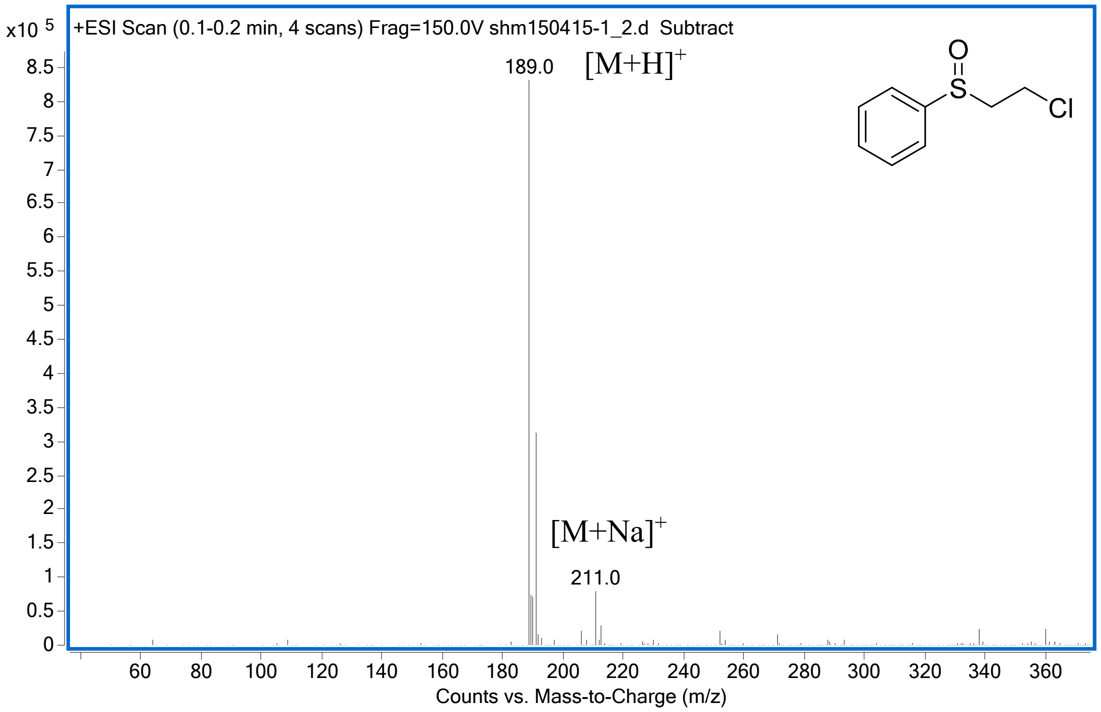

Figure S34. ESI-MS spectrum of 2-chloroethyl phenyl sulfoxide.

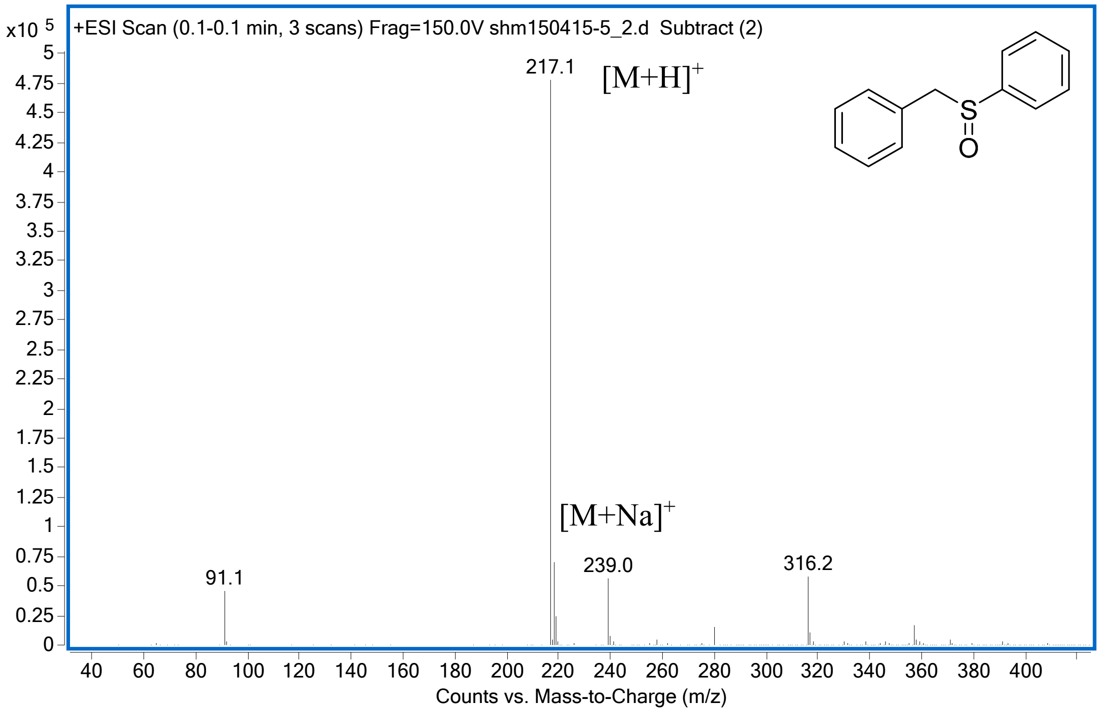

Figure S35. ESI-MS spectrum of benzyl phenyl sulfoxide.

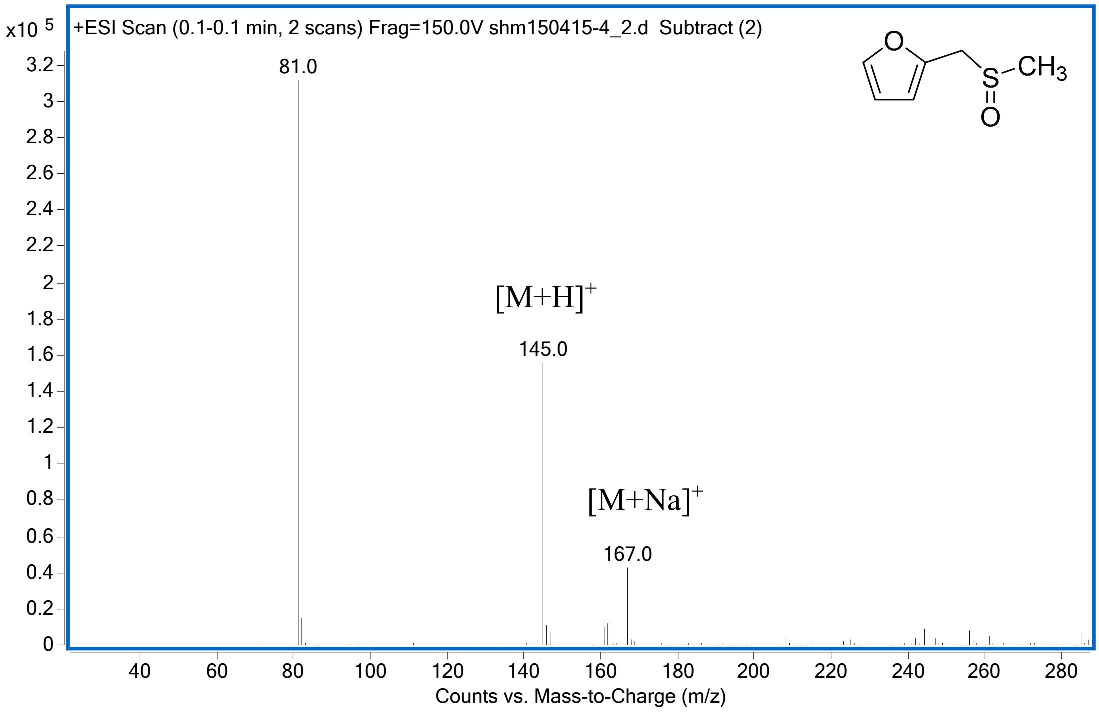

Figure S36. ESI-MS spectrum of furfuryl methyl sulfoxide.

6. Typical GC Spectra for the Product Sulfoxides

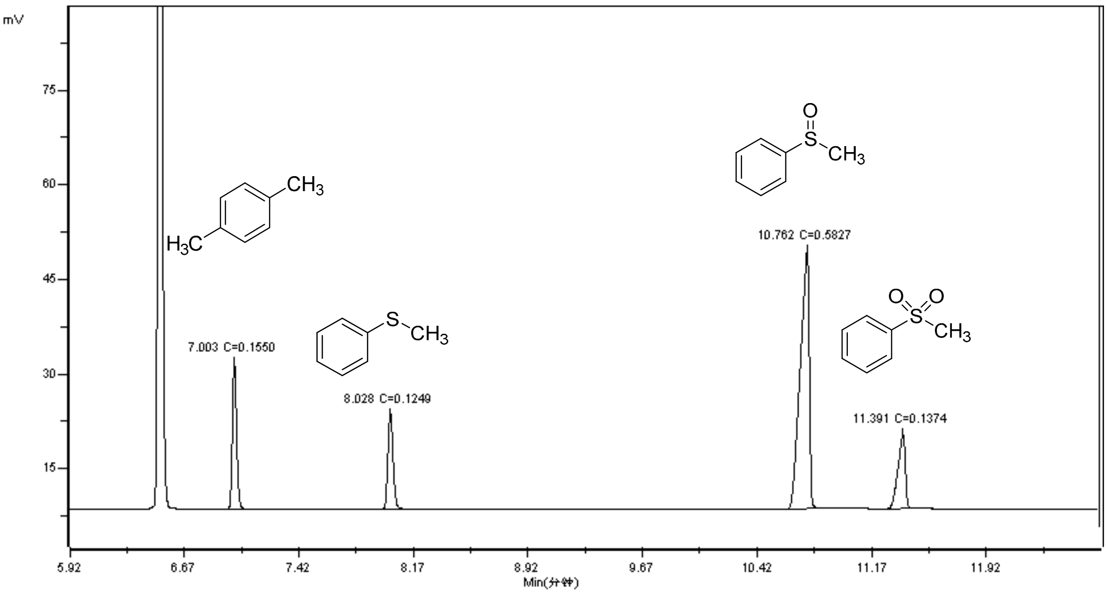

Figure S37. Typical qualitative spectrum.

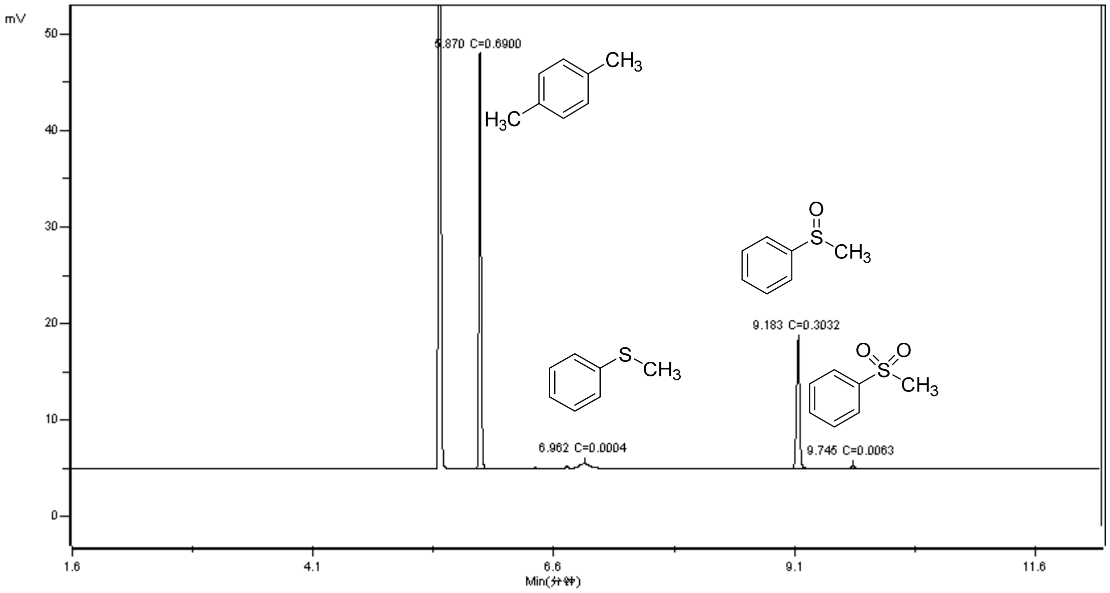

Figure S38. Typical quantitative spectrum.
